# Advances in the prevention and prenatal treatment of spina bifida

**DOI:** 10.15190/d.2026.1

**Published:** 2026-03-02

**Authors:** Zachary B. Sluzala, Katrina E. Furth

**Affiliations:** ^1^Charlotte Lozier Institute, Arlington, VA 22206, USA; ^2^The Catholic University of America, Psychology Department, Washington, DC 20064, USA

**Keywords:** Spina bifida, myelomeningocele, prenatal surgery, fetal surgery, fetal therapy, stem cells, biomaterials, neural tube defects.

## Abstract

Spina bifida is a neural tube defect (NTD) that arises when the neural tube fails to close properly during early development. This review focuses on myelomeningocele (MMC), the most common severe form of spina bifida, which often leads to motor and sensory impairments, including lower limb weakness or paralysis, as well as renal, urological, orthopedic, developmental, and psychosocial challenges. We explore the etiology, pathogenesis, prevention, diagnosis, and management of spina bifida, with a special emphasis on in-utero surgical repair. Over the past several decades, researchers and clinicians have made remarkable strides across all stages of care from prevention to postnatal outcomes. Widespread use of folic acid supplementation has significantly reduced the number of new cases. Advances in prenatal imaging and diagnostics now allow for earlier and more accurate detection, enabling timely intervention. In-utero surgical techniques continue to evolve, with innovative hybrid approaches that combine the strengths of open and minimally invasive methods. The momentum in this field shows no sign of slowing. Promising developments in stem cell therapy, biomaterials, robotic-assisted surgery, 3D printing, and enhanced imaging are redefining treatment goals in spina bifida. With each advance, clinicians gain better tools to improve outcomes for both mother and child, minimizing risks and maximizing long-term health and quality of life for both patients.

## SUMMARY

*1. Introduction*

*2. Spina Bifida Prognosis*

*3. Spina Bifida Prevention: Folic Acid and NTDs*

*4. Spina Bifida Diagnosis*

*5. In-Utero Treatment for Spina Bifida*

*6. In-Utero Treatment for Spina Bifida: Recent Advancements of Note*

*7. Unresolved Challenges*

*8. Conclusions*

## 1. Introduction

Spina bifida is a serious congenital condition caused by the incomplete closure of the neural tube during early embryonic development. Its prevalence varies widely across regions, with a global average ranging from 3.52 to 24.31 per 10,000 births.^1^ In the United States, spina bifida affects approximately 3.59 per 10,000 live births.^2^ Over the past three decades, early diagnosis and surgical innovation, especially prenatal intervention, have transformed the outlook for children with spina bifida. The landmark MOMS (Management of Myelomeningocele Study) trial and follow-up studies have demonstrated that fetal repair of myelomeningocele (MMC) can significantly improve motor outcomes and reduce the need for cerebrospinal fluid (CSF) shunting.^3,4^ Today, clinicians recognize the fetus as a patient in their own right, with a wide range of prenatal diagnostic tools and treatment options available. Advances in fetal medicine now allow healthcare teams not only to plan the timing, mode, and place of delivery—but in many cases, to treat the condition before birth. For families facing a spina bifida diagnosis, fetal surgery presents a hopeful alternative to termination and the potential to dramatically improve quality of life. This review synthesizes the current state of knowledge on open spina bifida, with a focus on prenatal surgery and outcome optimization.

### Spina Bifida, a Neural Tube Defect

**Figure 1:**
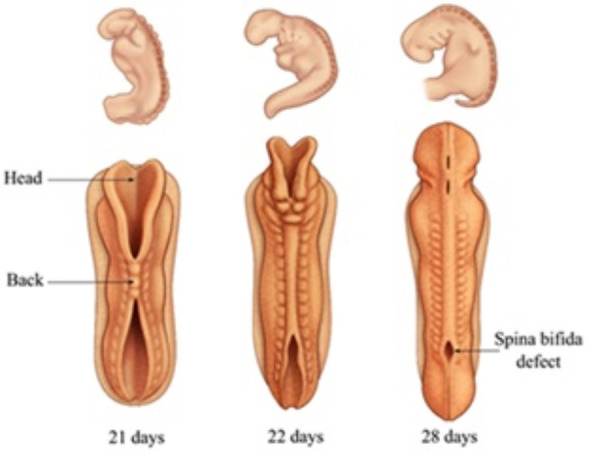
Folic acid makes major contributions to the development of the neural tube Neural tube formation is often complete before a woman knows that she is pregnant because she first expects her period about 15 days after conception. Countries such as the United States started adding 140 µg of folic acid to every 100 grams of enriched grains in the 1990s, to ensure that women have enough folic acid during the critical first month of pregnancy. This reduced nationwide cases of spina bifida by up to 2/3.^8^ Image reproduced from reference13, with permission.

 Spina bifida is a neural tube defect (NTD) that arises when the neural tube fails to close properly at the caudal (lower) end during early embryonic development.^5^ The neural plate, which formed from ectoderm around 17 days after conception,^6^ undergoes primary and secondary neurulation to form the brain and spinal cord. Primary neurulation creates a hollow tube through folding and separation from the surface around day 22, while secondary neurulation forms the lower spinal cord through the hollowing of a solid cell cord around day 26.^5,7,8^ Neural tube closure occurs in a zipper-like fashion across multiple regions, guided by signals from the notochord and surrounding tissues^9^ (see **Figure 1**). Neural tube defects are classified as open or closed depending on whether neural tissue is exposed to the intrauterine environment. In closed defects, neural elements are sealed off from the intrauterine environment; in open defects, neural elements, or a cyst containing neural elements, remain exposed to the amniotic fluid.^8^

In MMC, the most common severe form of spina bifida, neural elements protrude through an opening in the spine. In closed MMC, the meninges and spinal cord remain sealed off from the intrauterine environment inside a cyst. In contrast, in open MMC, the neural elements are directly exposed to amniotic fluid, which causes further injury.^8^ The commonly accepted “two-hit hypothesis” explains MMC pathogenesis: first a failure of neurulation, followed by progressive in-utero injury to exposed neural tissue.^10^ In the most severe, but rare form of spina bifida, myeloschisis (MS), the spinal cord is completely open on the back, forming a flat plate of neural tissue, or a “placode”.^11,12^ In both MMC and MS the caudal end of the spinal cord may remain fixed or tethered to the vertebral column, pulling the hindbrain into the spinal cord, known as hindbrain herniation, and blocking the flow of CSF resulting in hydrocephalus.^10^

## 2. Spina Bifida Prognosis

Myelomeningocele involves a wide range of motor, sensory, and developmental challenges. Children with MMC may experience lower limb weakness or paralysis, loss of sensation, and orthopedic conditions such as clubfoot, scoliosis, kyphosis (outward curvature of the spine), or hip dislocations.^11–13^ Many also face complications involving the renal and urological systems, including infections, and incontinence.^11–13^ Neurologically, MMC is often associated with hindbrain herniation, also known as Chiari II malformation, and hydrocephalus, which frequently requires cerebrospinal fluid diversion through ventriculoperitoneal shunting or endoscopic third ventriculostomy.^11–13^Some individuals may also experience seizures, along with cognitive and psychosocial difficulties.^11–13^Despite these challenges, survival rates have steadily improved, with 20-year survival ranging from 50% to 87% depending on the presence of hydrocephalus.^14^ Clinicians can now manage MMC more effectively thanks to advances in both prenatal and postnatal surgical techniques. Once viewed primarily as a life-threatening condition, MMC is now increasingly treated as a quality-of-life issue. The advent of fetal surgery has shifted treatment goals toward maximizing independence and developmental potential for affected children.^15^As medicine and technology have improved outcomes for children with spina bifida, one of the most powerful advances has been preventing the condition in the first place through folic acid supplementation.

## 3. Spina Bifida Prevention: Folic Acid and NTDs

Folic acid deficiency is a major contributor to the development of NTDs. Because folates cannot be synthesized from scratch, they must be obtained in the diet.^16^ Folic acid plays a critical role in synthesizing thymidylates, purines, and other components essential to the creation of DNA and RNA.^16^ In this role, folate plays a key role in cell proliferation and neurulation, processes vital for neural tube closure.^8^ This makes adequate folic acid intake especially important during early pregnancy, when the neural tube is forming. Pregnant women require five to ten times more folate than their age-matched peers.^17^

Early studies in the 1980s suggested that folic acid supplementation reduced the risk of NTDs, though initial trials were limited by small sample sizes^18^ and methodological concerns.^19^ In response, the UK Medical Research Council conducted a large, randomized controlled trial in 1991, which confirmed that a daily 4,000 μg dose of folic acid significantly reduced NTD recurrence.^20^ More recently, a large meta-analysis found that folic acid supplementation reduces the risk of NTDs by approximately two-thirds, although it did not demonstrate a clear protective effect for other congenital anomalies.^21^ Furthermore, a multicenter randomized controlled trial showed that supplementation with 4 mg of folic acid per day, compared with 0.4 mg, significantly reduced the occurrence of multiple birth defects, including NTDs.^22^ Globally, a daily intake of at least 400 μg of folic acid is recommended periconceptionally.^23^

Because neural tube closure often occurs before pregnancy is recognized (see **Figure 1**), relying solely on prenatal vitamin use may fail to protect many pregnancies. This recognition prompted widespread public health interventions, including mandatory folic acid fortification of cereal grain products in the United States beginning in 1998.^8^ Researchers predict that about 1300 fewer babies are given NTD diagnoses in the United States every year because of this folic acid fortification.^24^ Similar reductions in NTDs have been observed in other countries including Chile, Canada, Brazil and South Africa upon the introduction of folic acid enrichment programs.^25,26,27,28^ Currently, countries with folic acid enrichment programs have fewer cases of spina bifida (35.2 vs. 52.3 per 100,000 live births), even after accounting for stillbirth and termination of pregnancy.^1^ Despite these successes, not all countries have adopted fortification policies, leaving continued opportunities for global prevention.^1,8,29^While broader access to folic acid could prevent many cases of spina bifida worldwide, accurate and timely diagnosis remains crucial for babies who are still affected.

## 4. Spina Bifida Diagnosis

MMC is primarily diagnosed using prenatal ultrasound. Ultrasound interpretation is inherently subjective and dependent on fetal positioning; however, recent advancements have improved diagnostic accuracy. Computerized support software systems, such as those developed by Cengizler et al. allow for the algorithmic identification of the fetal spine, limiting subjectivity.^30,31^ Machine learning-based analyses have also been done to study the sonographer workflow, finding that each scan is a “non-ordered multistep process of anatomical structure acquisition” due in part to a need to take advantage of fetal position.^32^ These recent findings have helped standardize techniques while highlighting variables that can be improved upon. Ultrasound has also been shown to be a reliable diagnostic approach for MMC regardless of maternal body mass index (BMI),^33^ and cranial, spinal, ventricular, muscular, and post-surgical markers of MMC have been identified which further enhance diagnostic accuracy.^34-39^ In many cases, ultrasound is also often supplemented with molecular and biochemical testing, magnetic resonance imaging, and echocardiography ^39-47^ which help to confirm or clarify findings. Novel imaging and diagnostic techniques have also greatly advanced the capability of clinicians to identify these defects,^48^ including next-generation whole-exome sequencing technologies,^49^ which have led to the identification of several novel mutations associated with NTDs. ^50-57^ Advances in prenatal diagnosis made it possible not only to detect spina bifida earlier, but also to consider surgical repair before birth.

## 5. In-Utero Treatment for Spina Bifida

### History

Historically, open spina bifida has been managed with postnatal surgical repair, which involves removing exposed tissue, dissecting a connective strand at the base of the spinal cord called the filum terminale, and surgically folding the spinal cord into a tube through a process known as tubularization. While this approach protects the spinal cord and prevents further damage, it does not reverse neurological deficits. Recognizing the limitations of postnatal repair, researchers began exploring the potential of earlier intervention, specifically in-utero surgery, to improve outcomes by halting or reducing neurological injury before birth.

Initial experiments in the 1980s and 1990s demonstrated the feasibility of fetal repair in animal models, including primates,^58^ rats,^59,60^ pigs,^60^ and lambs.^61,62^These promising results paved the way for human trials. In 1997, Meuli-Simmen and colleagues reported the first successful technical attempt at in-utero MMC repair in humans.^63,64^ Early cases faced challenges: the first two fetal surgeries used a maternal skin graft to cover the exposed neural tissue - one baby survived to one year, while the other died due to extreme prematurity.^65^ However, rapid progress followed. By 1998 and 1999, teams at the Children’s Hospital of Philadelphia (CHOP) and Vanderbilt University reported improved outcomes with refined surgical techniques.^66-69^ Later, in 2003, Dr. Harrison’s team at the University of California, San Francisco reported their attempt at fetoscopic MMC repair.^70^ Complete fetoscopic repair was only accomplished in one case, with two other fetuses undergoing partial fetoscopic repair. Dr. Harrison’s team then temporarily abandoned the fetoscopic approach for the remaining 10 fetuses, opting instead to utilize an open approach.^70^ Efforts to optimize fetal surgical techniques culminated in the landmark Management of Myelomeningocele Study (MOMS) trial, published in 2011,^3^ which confirmed that prenatal surgery significantly improves motor outcomes and reduces the need for CSF shunting, marking a turning point in the standard of care. Notable clinical trials advancing prenatal spina bifida repair can be found in **Table 1**.

**Table 1:** Notable clinical trials advancing prenatal spina bifida repair

Clinical Trial	Outcomes	Year	Ref
Management of Myelomeningocele Study (MOMS) & Follow-up	Prenatal (open hysterotomy) repair is superior to postnatal repair in terms of shunt rate, reversal of hindbrain herniation, ambulation, and other metrics.	2011-2022	^3,71-77^
Cirurgia Endoscópica para Correção Antenatal da Meningomielocele (CECAM)	Minimally invasive fetoscopic repair is feasible, albeit with risks such as membrane rupture.	2016	^78^
Fetoscopic Meningomyelocele Repair Study (fMMC) & Follow-up	Hybrid laparotomy-assisted fetoscopic repair is feasible, with similar benefits to open repair and lowered risk of prematurity.	2017-2025	^79-81^
Fetoscopic NEOX Cord 1K® Spina Bifida Repair	Investigating feasibility of human umbilical cord patches for defect coverage.	Ongoing	^82^
Cellular Therapy for In Utero Repair of Myelomeningocele - The Cure Trial (CuRe)	Investigating application of stem cells to defect. Ongoing but thus far positive outcomes.	Ongoing	^83,84^

### The MOMS Trial & Open Hysterotomy Spina Bifida Repair

The MOMS Trial showed that prenatal surgery vastly improved the child’s mental and motor outcomes compared to postnatal surgery.^3^ In the study, clinicians utilized an open-hysterotomy prenatal closure approach, comparing outcomes to children treated postnatally. In this approach, a laparotomy incision is made to expose and exteriorize the uterus. A uterine incision is then made to expose the MMC, using ultrasound to guide the location of the hysterotomy. The neural placode is dissected and dropped into the spinal canal. The dura, skin, and uterus are then closed.^72^ During the MOMS Trial, participating centers coordinated with non-participating centers, who agreed not to perform

The MOMS Trial demonstrated remarkable results, and in fact was stopped early due to the efficacy of prenatal MMC repair over postnatal repair, particularly in terms of hindbrain herniation reversal and hydrocephalus. Prenatally repaired children required hydrocephalus management by CSF diversion via shunt placement at less than half the rate of postnatally repaired children (40% vs. 82%). Partial or total hindbrain herniation reversal was also markedly different, with 75% of prenatally repaired children showing no moderate or severe hindbrain herniation and 36% showing complete hindbrain herniation resolution, compared to 33% and 4% of postnatally repaired children, respectively. The incidence of brainstem kinking was also lower in prenatally repaired children (20% vs. 49%), as was abnormal 4^th^ ventricular location (46% vs. 72%) and rates of spinal cord cysts called syringomyelia (39% vs. 58%).^3^ An update was published outlining all 12-month outcome data and expanding upon the initially presented findings. The reduced need for CSF shunting was maintained (44% vs. 83.6%), and this follow-up data revealed that prenatally repaired children also needed fewer shunt revisions after placement (15.4% vs. 40.2%).^73^ Amazingly, even at the 5–10-year follow-up, prenatally repaired children required fewer shunts (49% vs. 85%) and fewer shunt revisions (23% vs. 60%), and the rate of total hindbrain herniation reversal had climbed and remained markedly improved (39% vs. 13%).^74^ Rates of syringomyelia also remained starkly different (59% vs. 81%). Most critically, neonatal death rates remained low and comparable between groups across this time range, with a 95% survival rate in the prenatal surgery group (5 total deaths of 91) and a 97% survival rate in the postnatal surgery group (3 total deaths of 92).^71,74^

As reduced lower body motor function and paralysis are particularly detrimental impacts of spina bifida, MOMS Trial investigators also longitudinally measured motor function improvements, and the results were similarly impressive. 44.8% of prenatally repaired children were walking independently at their 30-month follow-up exam, 27.6% were walking with the assistance of orthotics or devices, and only 27.6% were not walking. In marked contrast, the postnatally repaired group had rates of 23.9% (independent), 35.3% (assisted), and 40.9% (not walking).^75^ Prenatally repaired children also exhibited lower rates of leg-length discrepancy, and required fewer orthopedic treatments with casts or braces at both 12 and 30 months.^77^ In addition, prenatally repaired children demonstrated greater object manipulation, general locomotion and mobility, and stationary motor function than postnatally repaired children. In fact, these improvements in motor function were more often greater than expected and less often worse than expected based on anatomical measures, in comparison to postnatally repaired children.^3,75^ At the 5–10-year follow-up, these differences persisted, and prenatally repaired children were more likely to walk independently, less likely to require a wheelchair, and more likely to be capable of walking outdoors than their postnatally repaired counterparts.^71,74^ These physical improvements translated into greater self-care capabilities. Prenatally repaired children were more competent chewing and swallowing, using a fork, brushing their teeth, washing and drying their hands, removing their socks and shoes, and zipping, showcasing the real-world benefits to these children.^71^ Prenatally repaired children were even found to be taller than their postnatally repaired counterparts.^74^

Besides physical improvements, prenatally repaired children in the MOMS Trial exhibited several mental, developmental, and social improvements, particularly in comparison to the postnatally repaired children. Prenatally repaired children exhibited greater psychomotor development than those repaired postnatally,^3,75^ and greater verbal learning, nonverbal reasoning, and reading scores.^74^ In fact, on cognitive and neurological measures for which prenatally repaired children did not outperform postnatally repaired children, no robust differences were noted besides fine motor control.^74^ Socially and developmentally, prenatally repaired children experienced a higher quality of life with less negative impact on their families, with the differences being maintained through the 5–10-year follow-up.^74,76^

Outside of the MOMS Trial, comparisons of outcomes between postnatal and prenatal repair are still relatively limited.^85^ However, several non-MOMS studies have validated these findings, demonstrating similarly improved neonatal outcomes including a reduced need for a shunt or shunt revision, higher rates of hindbrain herniation resolution, improved neonatal functional level, and shorter length of stay in neonatal intensive care units than postnatal repair.^86-88^ Another study showed that fetal spina bifida repair may also help normalize cerebral blood flow.^89^ Similar benefits to ambulation have been shown in non-MOMS studies as well,^80,90,91^ with one study even showing prenatally repaired children participating in sports activities at later time points.^91^ Non-MOMS studies have found prenatally repaired children exhibit adaptive behavior, executive functioning abilities, neurocognitive outcomes, psychoeducational achievement, and memory scores within the age-matched population norms, albeit with delays or deficits, particularly for children with shunts.^91-93^

Open hysterotomy-based prenatal spina bifida surgery is not without its drawbacks, however (see **Table 2**). As with many other prenatal surgeries, open MMC repair is more strongly associated with preterm delivery, low birth weight, and other obstetric issues such as uterine rupture, membrane separation or rupture (including preterm premature rupture of membranes or PPROM), maternal pulmonary edema, placental abruption, and oligohydramnios (reduced amniotic fluid) than postnatal repair.^3,67,80,86,88,94,95^However, the risk of some of these complications is notably similar to the risk following classical c-section.^94,96,97^ Prenatal surgery has also been more strongly associated with certain perinatal complications, such as respiratory distress syndrome and bradycardia.^3,80^ Other findings, particularly on urological function post-repair, are mixed. Both MOMS and non-MOMS studies have shown positive findings, such as prenatally repaired children being more likely to void volitionally and have lower rates of urinary tract infection, but there are also negative findings such as a higher prevalence of bladder muscle overactivity compared to postnatally repaired children.^98,99^ As the field of fetal surgery continues to evolve, advancements will likely improve the outlook for mothers and children following open prenatal MMC repair.^100^

### Advances in Open Hysterotomy-Based Prenatal MMC Repair

Following the MOMS Trial, inclusion criteria for open prenatal MMC repair have expanded. Although high BMI was initially an exclusion factor, later studies have shown comparable perioperative outcomes in women with higher BMI.^101^ Access has also expanded to include women with gestational diabetes, certain fetal abnormalities, maternal infections and Rh alloimmunization,^4,102^ and discussion continues to be had about further loosening eligibility criteria.^103^ CHOP has also developed a new closure technique, which uses a needlepoint monopolar cautery to raise myofascial flaps, that has been shown to result in reduced rates of CSF diversion, more significant closure, reductions in hindbrain herniation and shunting, and overall improved results.^4,104^ Due to these technical modifications and increased experience, outcomes reported post-MOMS by CHOP have improved. As of 2017, PPROM rates had dropped from the 46% observed in MOMS to 32%, repair site dehiscence (rupture) rates had dropped from 13% to 3.6%, and pulmonary edema, the need for maternal blood transfusion, and severe preterm birth rates all dropped substantially as well.^4,88^

Even postnatal repair continues to improve. Intraoperative neurophysiological monitoring, which has been used extensively in spinal surgery already, has been recently shown to be useful in postnatal neonatal spina bifida repair as well.^105^

Several other approaches in addition to the open hysterotomy-based repair technique have also developed, which offer unique benefits over postnatal and open prenatal repair. These include percutaneous fetoscopic repair, laparotomy-assisted fetoscopic repair, and a percutaneous/mini-laparotomy fetoscopic technique.^78,81,106-111^ Anesthetics and analgesics are administered to both the mother and the fetus during all these procedures.^78,108,112^

### Percutaneous Fetoscopic Repair

Percutaneous fetoscopic repair of MMC is a minimally invasive alternative to open fetal surgery that has evolved significantly over time. The procedure involves inserting instruments through small punctures in the maternal abdomen and uterus, dissecting the neural placode, covering it with a patch, and closing the defect, all without a large uterine incision. Unlike open hysterotomy, which requires cesarean section, percutaneous fetoscopic repair allows for vaginal delivery, and has been associated with a lower risk of maternal complications such as uterine thinning, rupture, and pulmonary edema (see **Table 2**).^113-116^ However, percutaneous fetoscopy carries higher rates of placental abruption, PPROM, and earlier gestational age at delivery, contributing to complications like respiratory distress syndrome, site dehiscence, sepsis, and increased need for CSF diversion.^107,115-118^Despite these risks, some studies have reported better neurological outcomes, including higher rates of hindbrain herniation reversal, urinaryc ontinence, and independent walking compared to open repair.^115,118^

Technological refinements have improved outcomes over time. The technique has been adapted to close larger spinal defects with comparable success, and the gestational age window for surgery has been extended slightly later (to 27+6 weeks) without compromising outcomes, which helps reduce the risk of extreme prematurity.^119,120^ Most recently, hybrid surgical approaches have emerged, aiming to leverage the benefits of both open and fetoscopic techniques while limiting the drawbacks, reflecting the continued drive to optimize safety and effectiveness for both mother and child.

### Hybrid Approaches

The hybrid laparotomy-assisted fetoscopic approach, developed in part by Texas Children’s Fetal Center,^79^ combines elements of open and fetoscopic MMC repair. Surgeons begin with a maternal abdominal incision to partially exteriorize the uterus. The membranes are secured to the uterine wall, amniotic fluid is replaced with carbon dioxide, and a fetoscope is inserted. The neural placode is dissected and closed fetoscopically.^81,112^This hybrid repair offers several benefits over earlier techniques. Unlike open surgery, it allows for vaginal delivery and achieves higher gestational age at birth than both open and percutaneous repairs.^80,87,107,116,121-123^ Vaginal delivery following laparotomy-assisted fetoscopic repair has also been associated with shorter length of stay in neonatal intensive care units.^122^ While maternal complication rates (e.g., PPROM, edema, preeclampsia) are generally similar to open repair, laparotomy-assisted fetoscopic is associated with lower rates of uterine thinning or dehiscence.^80^ Neonatal complication rates are similarly very comparable.^80,121^ Though laparotomy-assisted fetoscopic repair takes longer than open repair and initially raised concerns about carbon dioxide use, studies have shown no adverse impact on fetal growth or long-term development.^124^ Importantly, when factors such as gestational age at delivery, age at time of outcome measurement, and presence of hydrocephalus treatment are controlled for, long term motor and neurodevelopmental outcomes are also comparable to open repair.^80,121,125,126^

Over time, technical refinements in the laparotomy-assisted fetoscopic approach have improved results. A multilayered closure technique reduced CSF leakage, skin dehiscence, and tethered cord, while improving rates of hindbrain herniation reversal.^80^ More recently, a California team introduced a “percutaneous/mini-laparotomy” hybrid approach, which uses a small incision and camera port for fetoscopic closure. Early results suggest a lower risk of prematurity than percutaneous repair and the possibility of vaginal delivery, though PPROM remains a challenge.^108^ Together, these innovations reflect the ongoing evolution of fetal surgery, balancing safety and precision while expanding treatment options for spina bifida.

As of 2017, ACOG and NAFTNet recommend providing women with a fetal MMC diagnosis the option to receive open fetal repair. They do not yet recommend fetoscopic fetal repair due to limited data.^127^ Although open fetal surgery is currently recommended by ACOG, emerging bioengineering strategies are introducing new tools and techniques that could further improve outcomes. Several surgical approaches are shown in **Table 2.**

**Table 2:** Benefits and drawbacks of different surgical approaches

Approach	Key Benefits	Key Drawbacks
Postnatal Repair	• Non-invasive (post-birth)• Decreased risk of maternal morbidity• Decreased logistical complexity	• Worst postnatal outcomes• Little reversal of neurological defects
Open Hysterotomy	• Well-established• Improved postnatal outcomes	• Invasive• Necessitates c-section delivery• Risk of maternal morbidity
Percutaneous Fetoscopic	• Least invasive• Allows vaginal birth• Improved postnatal outcomes	• More time-consuming• Increased risk of prematurity• Limited data
Laparotomy-Assisted Fetoscopic	• Less invasive• Allows vaginal birth• Improved postnatal outcomes• Lowest risk of prematurity among prenatal techniques	• More time-consuming• Limited data
Percutaneous/Mini-Laparotomy	• Less invasive• Allows vaginal birth• Lowered risk of prematurity	• More time-consuming• Limited data

## 6. In-Utero Treatment for Spina Bifida: Recent Advancements of Note

Additional improvements to open and fetoscopic approaches, as well as to postnatal repair procedures not outlined above have improved the outcomes and processes further. Modified closure techniques have also been tested since the MOMS trial. Two-layer closure (myofascial and skin) has been shown to improve watertightness and outcomes,^128^ and another more recently developed 3-port, 3-layer fetoscopic repair technique may further improve outcomes and lead to higher rates of watertight closure.^129,130^ MOMS3, a follow-up of MOMS participants in teen and young adult years, is also currently planned and enrolling study participants.^131^There are also numerous groundbreaking advancements being developed that make use of stem cells and bioengineered tissues, and which leverage other technological modalities such as robotics, 3D printing, and plastic surgery.

### Stem Cell and Biomaterials Based Approaches

Stem cells, tissue engineering, and biomaterials-based advances have continued to drive fetal spina bifida repair forward.^132-134^ In 2008, the concept of applying neural stem cells to spina bifida defects to aid in repair was validated in an animal model. Authors showed that application of stem cells to induced MMC defects in a lamb model led to the local production of neurotrophic factors.^135^ This approach has since been built upon with the use of early gestational placental mesenchymal stromal cells (PMSCs) in prenatal MMC repair. These PMSCs are isolated from placental chorionic villus tissue and can be subsequently expanded and banked.^136^ The cells can then be seeded into an extracellular matrix delivery vehicle which is applied to the defect. In mouse^137^ and sheep^138-143^ models, this approach has shown promising results. Now, a clinical trial is currently underway at UC Davis Health called the “CuRe Trial: Cellular Therapy for In Utero Repair of Myelomeningocele”, which uses the approach outlined above to complement fetal surgery for spina bifida treatment. Robbie, the first baby who received this treatment, moved her legs and wiggled her toes after birth, and is thus far in very great health. Robbie is now crawling and kicking, and other babies have undergone the same treatment as the trial continues.^83,84^ See **Supplemental video 1** (https://discoveriesjournals.org/art/Suppl/Supplemental%20Video1-CuReTrial.mp4) for an animation of the procedure used in the CuRe Trial.^144^

Another stem cell-based advancement in development is the use of transamniotic stem cell therapy (TRASCET)^145^as an alternative to open fetal MMC repair. TRASCET involves harnessing and augmenting biological roles of specific fetal stem cells – in the context of spina bifida, placental-, amniotic fluid-, or bone marrow-derived stem cells – to exert therapeutic benefits. Rat and rabbit models utilizing this approach have shown promising results, with higher rates of partial or complete defect coverage and reductions in hindbrain herniation rates.^146-151^Other stem cell-based advancements under investigation include differentiation of human induced pluripotent stem cells into neural crest stem cells for implantation into MMC defects, which has also been shown to be feasible in a lamb model;^152^ differentiation of amniotic fluid-derived stem cells into keratinocytes;^153^ and use of fibroblasts and keratinocytes to bioengineer lab-grown fetal skin, which has been demonstrated in lamb models as well.^154^

Other advancements in patch development and defect coverage are also being investigated. One such approach involves the use of basic fibroblast growth factors, delivered via an extracellular gelatin matrix for coverage of the spina bifida defect. In 2010, researchers at CHOP demonstrated preliminary success in adhering their growth factor-crosslinked hydrogel sponge scaffold to the MMC defect in a rat model, with evidence of tissue ingrowth and angiogenesis.^155^ They then went on to show equivalent effectiveness between this gelatin sponge and gelatin microspheres.^156^ More recently, in 2016, these researchers reported feasibility of this approach in a sheep model,^157^ complementing their previous work in rats. Another group at Yale is also investigating growth factor-based defect coverage and has shown success in rat models.^158^The feasibility of covering the defect using collagen-, small intestinal submucosa-, silicone, polypropylene or high-density polyethylene-, amniotic membrane-, biosynthetic cellulose-, and nanofiber-based scaffolds/patches, has also been shown in experimental animal models, and in some cases human patients.^110,159-171^Defect coverage using umbilical cord-derived patches has also been shown to be successful in both rat^172^ and sheep^173-177^ models and in human fetal spina bifida repair.^178^ There is currently an active clinical trial attempting to show feasibility of coverage of spina bifida defects with these patches as well.^82^

### Plastic Surgery, Robotic Surgery, and 3D Printed Materials

Outside of biomaterials, technological and engineering innovations have steadily enhanced prenatal MMC repair. In Zurich, researchers successfully demonstrated the first in-utero use of pedicled random pattern transposition flaps - which have been commonly used in adult and pediatric surgeries - for closing spinal defects, with minimal complications.^179^ Robotic-assisted fetal surgery is another promising frontier.^15,180^ Although not yet standard, multiple animal studies have shown the feasibility of robot-assisted endoscopic repair,^181-184^ including novel methods using customized magnetic catheters to improve precision.^184^ A major milestone came with the 2025 “Fetoscopic Robotic Open Spina Bifida Treatment” (FROST) study, which showcased the integration of robotics and 3D printing. Researchers created lifelike, 3D-printed uterine models, including a silicone fetus and placenta, to train surgeons in robot-assisted MMC repair. After 15–21 simulations, the surgeons could competently perform mock procedures.^185^ Possible use of 3D printed materials in training contexts such as these was discussed at a teaching session at the 2018 EuroCMR/SCMR joint congress, at which clinicians agreed that there were potential applications for these materials.^186^ But use cases have expanded beyond training, and 3D printing is now being used to assess patch requirements and build patient-specific models for surgical planning.^187,188^ These advancements highlight how technology is driving safer, more effective, and increasingly personalized approaches to fetal spina bifida repair. The fruits of this can be seen not only in the United States, but internationally as well.

### International Adoption

International adoption of prenatal spina bifida repair has also progressed, though variability exists in terms of inclusion and diagnostic criteria, surgical techniques, perioperative management strategies, and neonatal resuscitation practices.^189-192^ Despite this variability, there has also been increasing collaboration between prenatal MMC repair centers in different countries, and the development of more multidisciplinary approaches and teams.^193^ Several non-U.S. countries are continuing to expand access to prenatal MMC repair and have seen improvements in outcomes over time, including (but certainly not limited to) Germany,^194-198^ Brazil,^78,111^ Taiwan,^199^ Canada,^200,201^ France,^202,203^ and Israel.^204^As prenatal repair of MMC expands to more countries and healthcare systems, it also raises important ethical questions about risk, access, and responsibility.

## 7. Unresolved Challenges

### Limitations of Recent Advances

The advances outlined above have resulted in remarkable progress. However, they are not without limitations. Some, but not all the new techniques and technologies have been validated in humans, raising questions about translatability. For those which have shown applicability in human patients, limited data and small sample sizes of existing trials and studies must also be considered. As additional data are collected demonstrating safety and efficacy, it is likely that these novel interventions will need to roll out in waves. Eligibility criteria will start relatively narrow and expand over time, as has been the case for prenatal surgical approaches outlined above. Regulatory hurdles should also be considered and foreseen. These approaches not only utilize biomaterials but also involve treatment of the most vulnerable group of people, unborn children, and therefore will understandably be met with scrutiny by regulatory agencies. While this scrutiny is crucial in ensuring that pregnant women and their children are protected, it will likely slow progress towards therapeutic advancements. Unresolved challenges exist not only pertaining to recent advances, but also to existing treatments and their availability.

### Limited Domestic Access to Prenatal MMC Repair

Although adoption of in-utero MMC repair has grown in the United States, access remains limited. A 2024 national survey found that only 31% of U.S. hospitals offered both prenatal and postnatal MMC repair, while the majority provided postnatal repair only.^205^One way by which access could be increased would be expansion of insurance coverage to support maternal travel to specialized centers offering prenatal MMC repair. Given the compelling evidence that prenatal repair significantly improves neurodevelopmental outcomes and mobility while reducing the long-term need for interventions such as shunting and catheterization, these benefits would translate not only to enhanced quality of life for affected children and their families but also to substantial healthcare cost savings over a lifetime.

### Maternal Complications vs. Fetal Benefit

The main drawback of in-utero spina bifida repair is the incidence of negative maternal outcomes, as outlined above. The ethics of fetal surgery have been discussed at greater length elsewhere,^206,207^ but frequently discussed considerations include the weighing of risks to the mother against the benefits to the fetus. As with many other fetal surgical interventions, fetal MMC repair comes with risks of preterm birth, ruptured membranes, dehiscence, and other obstetric complications. Though as noted, the risk of some of these complications such as rupture are similar to typical c-section rates.^94,96,97^ Each of the approaches outlined above come with benefits and drawbacks, with some (such as the percutaneous/mini-laparotomy approach) having much lower risks of uterine dehiscence, and others (such as the laparotomy-assisted fetoscopic approach) having lower risks of premature birth. As these techniques continue to develop further, it is possible that new, hybrid approaches will be able to further limit drawbacks while continuing to draw on the strengths of different techniques. However, these obstetric complications must also be viewed considering the reality that during a pregnancy, doctors and surgeons are treating two patients – the mother and her unborn child.^208-210^ In the words of the “Father of Fetal Surgery” himself, “The fetus is no longer a medical recluse hidden inside an opaque womb. The fetus is a patient with problems that cannot only be examined by an array of prenatal tests, but also can be actively managed by arranging the timing, mode, and place of delivery. In a few cases, the fetal problem can even be treated before birth”.^210^ For this second patient, prenatal surgery offers an option besides termination and a markedly improved quality of life.

## 8. Conclusions

Our understanding of spina bifida has greatly improved over the past several decades, with advances being made from prevention to diagnosis and management. A better grasp on the preventative roles of folic acid has allowed fewer cases of spina bifida to manifest in the first place. This highlights that clinicians worldwide should encourage folic acid supplementation prior to and during pregnancy. Improved imaging and diagnostics have allowed for more rapid and accurate identification of those cases which do manifest. Clinicians are now equipped with a multitude of techniques to repair the defect in utero, as new, hybrid approaches continue to develop to harness the benefits and limit the drawbacks of each. In practice, the findings of this review and the broader literature will inform the selection of surgical techniques by clinicians based upon each woman’s circumstances and risk profile. As the field moves forward, improvements will continue to be made. Stem cells and other biomaterials, robotic surgeries, 3D printing, improved imaging and diagnostic markers, and other refinements in technology and technique have consistently pushed the limits of what is possible. Further work is needed to expand treatment availability so that more expecting mothers and their unborn children can access the fruits of this advancing field and pursue positive outcomes for both patients.

## Key Points

- *Clinicians now recognize the fetus as a patient in their own right.*

- *Folic acid supplementation plays an important role in spina bifida prevention worldwide.*

- *Prenatal surgical approaches for myelomeningocele (MMC) have evolved and continue to evolve, expanding treatment options and improving outcomes for both mother and child.*

## Author Contributions

Conceptualization, Z.B.S.; writing - original draft preparation Z.B.S.; writing - review and editing, Z.B.S. and K.E.F.; figure preparation, K.E.F.; project administration, Z.B.S. All authors have read and agreed to the published version of the manuscript.

## Publisher’s note

All claims expressed in this article are solely those of the authors and do not necessarily represent those of their affiliated organizations, or those of the publisher, the editors and the reviewers. Any product that may be evaluated in this article, or claim that may be made by its manufacturer, is not guaranteed or endorsed by the publisher.

## References

[1] Atta Callie A M, Fiest Kirsten M, Frolkis Alexandra D, Jette Nathalie, Pringsheim Tamara, St Germaine-Smith Christine, Rajapakse Thilinie, Kaplan Gilaad G, Metcalfe Amy (2016). Global Birth Prevalence of Spina Bifida by Folic Acid Fortification Status: A Systematic Review and Meta-Analysis.. American journal of public health.

[2] Stallings Erin B, Isenburg Jennifer L, Rutkowski Rachel E, Kirby Russell S, Nembhard Wendy N, Sandidge Theresa, Villavicencio Stephan, Nguyen Hoang H, McMahon Daria M, Nestoridi Eirini, Pabst Laura J (2024). National population-based estimates for major birth defects, 2016-2020.. Birth defects research.

[3] Adzick N Scott, Thom Elizabeth A, Spong Catherine Y, Brock John W, Burrows Pamela K, Johnson Mark P, Howell Lori J, Farrell Jody A, Dabrowiak Mary E, Sutton Leslie N, Gupta Nalin, Tulipan Noel B, D'Alton Mary E, Farmer Diana L (2011). A randomized trial of prenatal versus postnatal repair of myelomeningocele.. The New England journal of medicine.

[4] Moldenhauer Julie S, Adzick N Scott (2017). Fetal surgery for myelomeningocele: After the Management of Myelomeningocele Study (MOMS).. Seminars in fetal & neonatal medicine.

[5] Singh Ranbir, Munakomi Sunil (2025). Embryology, Neural Tube.

[6] Torchia, Persaud (2024). The Developing Human: Clinically Oriented Embryology. 12th edition..

[7] Shaikh Fouziya, Sanadhya Mallica, Kaleem Safa, Verma Tiya, Jayaraj Richard L, Ahmad Faizan (2025). Critical appraisal on neural tube defects and their complexities.. Pediatrics and neonatology.

[8] Ledet Iii Lloyd F, Plaisance Connor J, Daniel Charles P, Wagner Maxwell J, Alvarez Ivan, Burroughs Caroline R, Rieger Ross, Siddaiah Harish, Ahmadzadeh Shahab, Shekoohi Sahar, Kaye Alan D, Varrassi Giustino (2024). Spina Bifida Prevention: A Narrative Review of Folic Acid Supplements for Childbearing Age Women.. Cureus.

[9] Isaković Jasmina, Šimunić Iva, Jagečić Denis, Hribljan Valentina, Mitrečić Dinko (2022). Overview of Neural Tube Defects: Gene-Environment Interactions, Preventative Approaches and Future Perspectives.. Biomedicines.

[10] Aydin Emrah, Peiro Jose Luis, Habli Monuira (2025). Closing the Gap: Prenatal Repair and the Reimagined Future of Spina Bifida.. Clinical obstetrics and gynecology.

[11] Karsonovich, Alruwaili, Das (2025). Myelomeningocele.

[12] Copp Andrew J, Adzick N Scott, Chitty Lyn S, Fletcher Jack M, Holmbeck Grayson N, Shaw Gary M (2015). Spina bifida.. Nature reviews. Disease primers.

[13] Parvin Afroza, Hasan Md Mahmudul (2023). An Overview of Spina Bifida. Open Journal of Orthopedics.

[14] Tennant Peter W G, Pearce Mark S, Bythell Mary, Rankin Judith (2010). 20-year survival of children born with congenital anomalies: a population-based study.. Lancet (London, England).

[15] Boswell Timothy C., Ahn Edward S., Ruano Rodrigo, Gargollo Patricio C. (2020). Robotic Fetal Surgery: The Next Frontier?. Minimally Invasive and Robotic-Assisted Surgery in Pediatric Urology.

[16] Leung Kit-Yi, Pai Yun Jin, Chen Qiuying, Santos Chloe, Calvani Enrica, Sudiwala Sonia, Savery Dawn, Ralser Markus, Gross Steven S, Copp Andrew J, Greene Nicholas D E (2017). Partitioning of One-Carbon Units in Folate and Methionine Metabolism Is Essential for Neural Tube Closure.. Cell reports.

[17] Sato Kohji (2020). Why is folate effective in preventing neural tube closure defects?. Medical hypotheses.

[18] Laurence K M, James N, Miller M H, Tennant G B, Campbell H (1981). Double-blind randomised controlled trial of folate treatment before conception to prevent recurrence of neural-tube defects.. British medical journal (Clinical research ed.).

[19] Smithells R W, Nevin N C, Seller M J, Sheppard S, Harris R, Read A P, Fielding D W, Walker S, Schorah C J, Wild J (1983). Further experience of vitamin supplementation for prevention of neural tube defect recurrences.. Lancet (London, England).

[20] (1991). Prevention of neural tube defects: results of the Medical Research Council Vitamin Study. MRC Vitamin Study Research Group.. Lancet (London, England).

[21] De-Regil Luz Maria, Peña-Rosas Juan Pablo, Fernández-Gaxiola Ana C, Rayco-Solon Pura (2015). Effects and safety of periconceptional oral folate supplementation for preventing birth defects.. The Cochrane database of systematic reviews.

[22] Bortolus Renata, Filippini Francesca, Cipriani Sonia, Trevisanuto Daniele, Cavallin Francesco, Zanconato Giovanni, Somigliana Edgardo, Cesari Elena, Mastroiacovo Pierpaolo, Parazzini Fabio (2021). Efficacy of 4.0 mg versus 0.4 mg Folic Acid Supplementation on the Reproductive Outcomes: A Randomized Controlled Trial.. Nutrients.

[23] Gomes Sandra, Lopes Carla, Pinto Elisabete (2016). Folate and folic acid in the periconceptional period: recommendations from official health organizations in thirty-six countries worldwide and WHO.. Public health nutrition.

[24] Williams Jennifer, Mai Cara T, Mulinare Joe, Isenburg Jennifer, Flood Timothy J, Ethen Mary, Frohnert Barbara, Kirby Russell S (2015). Updated estimates of neural tube defects prevented by mandatory folic Acid fortification - United States, 1995-2011.. MMWR. Morbidity and mortality weekly report.

[25] Sayed Abdul-Rauf, Bourne David, Pattinson Robert, Nixon Jo, Henderson Bertram (2008). Decline in the prevalence of neural tube defects following folic acid fortification and its cost-benefit in South Africa.. Birth defects research. Part A, Clinical and molecular teratology.

[26] Rodrigues Viviane Belini, Silva Everton Nunes da, Dos Santos André Marques, Santos Leonor Maria Pacheco (2023). Prevented cases of neural tube defects and cost savings after folic acid fortification of flour in Brazil.. PloS one.

[27] De Wals Philippe, Tairou Fassiatou, Van Allen Margot I, Uh Soo-Hong, Lowry R Brian, Sibbald Barbara, Evans Jane A, Van den Hof Michiel C, Zimmer Pamela, Crowley Marian, Fernandez Bridget, Lee Nora S, Niyonsenga Theophile (2007). Reduction in neural-tube defects after folic acid fortification in Canada.. The New England journal of medicine.

[28] López-Camelo Jorge S, Castilla Eduardo E, Orioli Iêda M (2010). Folic acid flour fortification: impact on the frequencies of 52 congenital anomaly types in three South American countries.. American journal of medical genetics. Part A.

[29] Yacob Alex, Carr Christopher J, Foote Jake, Scullen Tyler, Werner Cassidy, Mathkour Mansour, Bui Cuong J, Dumont Aaron S (2021). The Global Burden of Neural Tube Defects and Disparities in Neurosurgical Care.. World neurosurgery.

[30] Cengizler Çağlar, Ün M Kerem, Büyükkurt Selim (2020). A Nature-Inspired Search Space Reduction Technique for Spine Identification on Ultrasound Samples of Spina Bifida Cases.. Scientific reports.

[31] Cengizler Caglar, Kerem Ün M, Buyukkurt Selim (2021). A novel evolutionary method for spine detection in ultrasound samples of spina bifida cases.. Computer methods and programs in biomedicine.

[32] Drukker L, Sharma H, Karim J N, Droste R, Noble J A, Papageorghiou A T (2022). Clinical workflow of sonographers performing fetal anomaly ultrasound scans: deep-learning-based analysis.. Ultrasound in obstetrics & gynecology : the official journal of the International Society of Ultrasound in Obstetrics and Gynecology.

[33] Barnes Katherine S, Singh Sumit, Barkley Ariana, Lepard Jacob, Hopson Betsy, Cawyer Chase R, Blount Jeffrey P, Rocque Brandon G (2022). Determination of anatomic level of myelomeningocele by prenatal ultrasound.. Child's nervous system : ChNS : official journal of the International Society for Pediatric Neurosurgery.

[34] Zhu Xia, Zhao Sheng, Yang Xiaohong, Feng Qian, Zhang Xiaoyan, Yang Fan, Chen Xinlin (2021). First-Trimester Cranial Ultrasound Markers of Open Spina Bifida.. Journal of ultrasound in medicine : official journal of the American Institute of Ultrasound in Medicine.

[35] Ungureanu Delia Roxana, Comănescu Maria Cristina, Istrate-Ofiţeru Anca-Maria, Zorilă George-Lucian, Drăgușin Roxana Cristina, Iliescu Dominic Gabriel (2023). Open Spina Bifida: The Role of Ultrasound Markers in the First Trimester and Morphopathology Correlation.. Current health sciences journal.

[36] Volpe Nicola, Bovino Alessandra, Di Pasquo Elvira, Corno Enrico, Taverna Michela, Valentini Beatrice, Dall'Asta Andrea, Brawura-Biskupsi-Samaha Robert, Ghi Tullio (2024). First-trimester ultrasound of the cerebral lateral ventricles in fetuses with open spina bifida: a retrospective cohort study.. American journal of obstetrics & gynecology MFM.

[37] Milani Hérbene José Figuinha, de Sá Barreto Enoch Quinderé, Araujo Júnior Edward, Cavalheiro Sérgio, Barbosa Maurício Mendes, Moron Antonio Fernandes (2022). Measurement of the Area and Circumference of the Leg: Preliminary Results of a New Method for Estimating Leg Muscle Trophism in Fetuses With Open Lumbosacral Spina Bifida.. Journal of ultrasound in medicine : official journal of the American Institute of Ultrasound in Medicine.

[38] Barreto Enoch Quinderé de Sá, Cavalheiro Sérgio, Milani Herbene José Figuinha, Barbosa Maurício Mendes, Araujo Júnior Edward, Nardozza Luciano Marcondes Machado, Moron Antonio Fernandes (2018). Cerebellar herniation demonstrated by the occipitum-dens line: Ultrasonography assessment of normal fetuses, fetuses with myelomeningocele, and fetuses that underwent antenatal myelomeningocele surgery.. Prenatal diagnosis.

[39] Yin Jiao, Wang Yan, Wang Sihong, Li Gang, Gu Hui, Chen Lizhu (2024). Research progress on ultrasound and molecular markers for prenatal diagnosis of neural tube defects.. Heliyon.

[40] Chao An-Shine, Jhang Lan-Sin, Hsieh Peter Ching-Chang (2024). Prenatal Diagnosis and Outcomes of Cervical Meningocele and Myelomeningocele.. Journal of medical ultrasound.

[41] Di Mascio Daniele, Greco Francesca, Rizzo Giuseppe, Khalil Asma, Buca Danilo, Sorrentino Felice, Vasciaveo Lorenzo, Greco Pantaleo, Nappi Luigi, D'Antonio Francesco (2021). Diagnostic accuracy of prenatal ultrasound in identifying the level of the lesion in fetuses with open spina bifida: A systematic review and meta-analysis.. Acta obstetricia et gynecologica Scandinavica.

[42] Nagaraj Usha D, Bierbrauer Karin S, Stevenson Charles B (2024). Imaging Fetal Spine Malformations in the Context of In Utero Surgery.. Magnetic resonance imaging clinics of North America.

[43] Nagaraj Usha D, Bierbrauer Karin S, Stevenson Charles B, Peiro Jose L, Lim Foong Yen, Habli Mounira A, Kline-Fath Beth M (2020). Prenatal and postnatal MRI findings in open spinal dysraphism following intrauterine repair via open versus fetoscopic surgical techniques.. Prenatal diagnosis.

[44] Tang Xing, Bai Guoyan, Wang Hong, Guo Fan, Yin Hong (2022). A comparison of the accuracy of fetal magnetic resonance imaging and ultrasonography for the diagnosis of fetal congenital malformations of the spine and spinal cord.. Prenatal diagnosis.

[45] Garel Juliette, Rossi Andrea, Blondiaux Eléonore, Cassart Marie, Hoffmann Chen, Garel Catherine (2023). Prenatal imaging of the normal and abnormal spinal cord: recommendations from the Fetal Task Force of the European Society of Paediatric Radiology (ESPR) and the European Society of Neuroradiology (ESNR) Pediatric Neuroradiology Committee. Pediatric Radiology.

[46] Meller César, Covini Delfina, Aiello Horacio, Izbizky Gustavo, Portillo Medina Santiago, Otaño Lucas (2021). Update on prenatal diagnosis and fetal surgery for myelomeningocele.. Archivos argentinos de pediatria.

[47] Walter Uwe (2025). What can fetal neurosonography reveal about the future of an unborn child?. Ultraschall in der Medizin (Stuttgart, Germany : 1980).

[48] Royal Charis, Chertin Leon, Alfawzan Mohammed, Killian Mary Elaine (2025). Novel Techniques in Antenatal Imaging of Spinal Dysraphisms.. Current urology reports.

[49] Lei Yunping, Finnell Richard H (2015). New Techniques for the Study of Neural Tube Defects. Advanced Techniques in Biology &amp; Medicine.

[50] Lemay Philippe, De Marco Patrizia, Traverso Monica, Merello Elisa, Dionne-Laporte Alexandre, Spiegelman Dan, Henrion Édouard, Diallo Ousmane, Audibert François, Michaud Jacques L, Cama Armando, Rouleau Guy A, Kibar Zoha, Capra Valeria (2019). Whole exome sequencing identifies novel predisposing genes in neural tube defects.. Molecular genetics & genomic medicine.

[51] Lemay Philippe, De Marco Patrizia, Emond Alexandre, Spiegelman Dan, Dionne-Laporte Alexandre, Laurent Sandra, Merello Elisa, Accogli Andrea, Rouleau Guy A, Capra Valeria, Kibar Zoha (2017). Rare deleterious variants in GRHL3 are associated with human spina bifida.. Human mutation.

[52] Lemay Philippe, Guyot Marie-Claude, Tremblay Élizabeth, Dionne-Laporte Alexandre, Spiegelman Dan, Henrion Édouard, Diallo Ousmane, De Marco Patrizia, Merello Elisa, Massicotte Christine, Désilets Valérie, Michaud Jacques L, Rouleau Guy A, Capra Valeria, Kibar Zoha (2015). Loss-of-function de novo mutations play an important role in severe human neural tube defects.. Journal of medical genetics.

[53] Azzarà Alessia, Rendeli Claudia, Crivello Anna Maria, Brugnoletti Fulvia, Rumore Roberto, Ausili Emanuele, Sangiorgi Eugenio, Gurrieri Fiorella (2021). Identification of new candidate genes for spina bifida through exome sequencing.. Child's nervous system : ChNS : official journal of the International Society for Pediatric Neurosurgery.

[54] Lei Yunping, Zhu Huiping, Duhon Cody, Yang Wei, Ross M Elizabeth, Shaw Gary M, Finnell Richard H (2013). Mutations in planar cell polarity gene SCRIB are associated with spina bifida.. PloS one.

[55] Lei Yunping, Kim Sung-Eun, Chen Zhongzhong, Cao Xuanye, Zhu Huiping, Yang Wei, Shaw Gary M, Zheng Yufang, Zhang Ting, Wang Hong-Yan, Finnell Richard H (2019). Variants identified in PTK7 associated with neural tube defects.. Molecular genetics & genomic medicine.

[56] Robinson Alexis, Escuin Sarah, Doudney Kit, Vekemans Michel, Stevenson Roger E, Greene Nicholas D E, Copp Andrew J, Stanier Philip (2012). Mutations in the planar cell polarity genes CELSR1 and SCRIB are associated with the severe neural tube defect craniorachischisis.. Human mutation.

[57] Beaumont Marie, Akloul Linda, Carré Wilfrid, Quélin Chloé, Journel Hubert, Pasquier Laurent, Fradin Mélanie, Odent Sylvie, Hamdi-Rozé Houda, Watrin Erwan, Dupé Valérie, Dubourg Christèle, David Véronique (2019). Targeted panel sequencing establishes the implication of planar cell polarity pathway and involves new candidate genes in neural tube defect disorders.. Human genetics.

[58] Michejda Maria (1984). Intrauterine Treatment of Spina Bifida: Primate Model. European Journal of Pediatric Surgery.

[59] Heffez D S, Aryanpur J, Hutchins G M, Freeman J M (1990). The paralysis associated with myelomeningocele: clinical and experimental data implicating a preventable spinal cord injury.. Neurosurgery.

[60] Heffez D S, Aryanpur J, Rotellini N A, Hutchins G M, Freeman J M (1993). Intrauterine repair of experimental surgically created dysraphism.. Neurosurgery.

[61] Meuli M, Meuli-Simmen C, Hutchins G M, Yingling C D, Hoffman K M, Harrison M R, Adzick N S (1995). In utero surgery rescues neurological function at birth in sheep with spina bifida.. Nature medicine.

[62] Meuli M, Meuli-Simmen C, Yingling C D, Hutchins G M, Timmel G B, Harrison M R, Adzick N S (1996). In utero repair of experimental myelomeningocele saves neurological function at birth.. Journal of pediatric surgery.

[63] Meuli M, Meuli-Simmen C, Hutchins G M, Seller M J, Harrison M R, Adzick N S (1997). The spinal cord lesion in human fetuses with myelomeningocele: implications for fetal surgery.. Journal of pediatric surgery.

[64] Meuli-Simmen C, Meuli M, Adzick N S, Harrison M R (1997). Latissimus dorsi flap procedures to cover myelomeningocele in utero: a feasibility study in human fetuses.. Journal of pediatric surgery.

[65] Bruner J P, Tulipan N E, Richards W O (1997). Endoscopic coverage of fetal open myelomeningocele in utero.. American journal of obstetrics and gynecology.

[66] Adzick N S, Sutton L N, Crombleholme T M, Flake A W (1998). Successful fetal surgery for spina bifida.. Lancet (London, England).

[67] Bruner J P, Tulipan N, Paschall R L, Boehm F H, Walsh W F, Silva S R, Hernanz-Schulman M, Lowe L H, Reed G W (1999). Fetal surgery for myelomeningocele and the incidence of shunt-dependent hydrocephalus.. JAMA.

[68] Sutton L N, Adzick N S, Bilaniuk L T, Johnson M P, Crombleholme T M, Flake A W (1999). Improvement in hindbrain herniation demonstrated by serial fetal magnetic resonance imaging following fetal surgery for myelomeningocele.. JAMA.

[69] Tulipan N, Hernanz-Schulman M, Bruner J P (1998). Reduced hindbrain herniation after intrauterine myelomeningocele repair: A report of four cases.. Pediatric neurosurgery.

[70] Farmer Diana l. (2003). In utero repair of myelomeningocele. Archives of Surgery.

[71] Houtrow Amy J, MacPherson Cora, Jackson-Coty Janet, Rivera Monica, Flynn Laura, Burrows Pamela K, Adzick N Scott, Fletcher Jack, Gupta Nalin, Howell Lori J, Brock John W, Lee Hanmin, Walker William O, Thom Elizabeth A (2021). Prenatal Repair and Physical Functioning Among Children With Myelomeningocele: A Secondary Analysis of a Randomized Clinical Trial.. JAMA pediatrics.

[72] Adzick N. Scott, Thom Elizabeth A., Spong Catherine Y., Brock John W., Burrows Pamela K., Johnson Mark P., Howell Lori J., Farrell Jody A., Dabrowiak Mary E., Sutton Leslie N., Gupta Nalin, Tulipan Noel B., D'Alton Mary E., Farmer Diana L. (2011). A Randomized Trial of Prenatal versus Postnatal Repair of Myelomeningocele. New England Journal of Medicine.

[73] Tulipan Noel, Wellons John C, Thom Elizabeth A, Gupta Nalin, Sutton Leslie N, Burrows Pamela K, Farmer Diana, Walsh William, Johnson Mark P, Rand Larry, Tolivaisa Susan, D'alton Mary E, Adzick N Scott (2015). Prenatal surgery for myelomeningocele and the need for cerebrospinal fluid shunt placement.. Journal of neurosurgery. Pediatrics.

[74] Houtrow Amy J, Thom Elizabeth A, Fletcher Jack M, Burrows Pamela K, Adzick N Scott, Thomas Nina H, Brock John W, Cooper Timothy, Lee Hanmin, Bilaniuk Larissa, Glenn Orit A, Pruthi Sumit, MacPherson Cora, Farmer Diana L, Johnson Mark P, Howell Lori J, Gupta Nalin, Walker William O (2020). Prenatal Repair of Myelomeningocele and School-age Functional Outcomes.. Pediatrics.

[75] Farmer Diana L, Thom Elizabeth A, Brock John W, Burrows Pamela K, Johnson Mark P, Howell Lori J, Farrell Jody A, Gupta Nalin, Adzick N Scott (2018). The Management of Myelomeningocele Study: full cohort 30-month pediatric outcomes.. American journal of obstetrics and gynecology.

[76] Antiel Ryan M, Adzick N Scott, Thom Elizabeth A, Burrows Pamela K, Farmer Diana L, Brock John W, Howell Lori J, Farrell Jody A, Houtrow Amy J (2016). Impact on family and parental stress of prenatal vs postnatal repair of myelomeningocele.. American journal of obstetrics and gynecology.

[77] Swarup Ishaan, Talwar Divya, Howell Lori J, Adzick N Scott, Horn Bernard David (2022). Orthopaedic outcomes of prenatal versus postnatal repair of myelomeningocele.. Journal of pediatric orthopedics. Part B.

[78] Pedreira Denise A L, Zanon Nelci, Nishikuni Koshiro, Moreira de Sá Renato A, Acacio Gregório L, Chmait Ramen H, Kontopoulos Eftichia V, Quintero Rubén A (2016). Endoscopic surgery for the antenatal treatment of myelomeningocele: the CECAM trial.. American journal of obstetrics and gynecology.

[79] (2025). Fetoscopic Meningomyelocele Repair Study (fMMC). National Library of Medicine. ClinicalTrials.gov..

[80] Sanz-Cortes Magdalena, Whitehead William E, Johnson Rebecca M, Aldave Guillermo, Castillo Heidi, Desai Nilesh K, Donepudi Roopali, Joyeux Luc, King Alice, Kralik Stephen F, Lepard Jacob, Mann David G, McClugage Samuel G, Nassr Ahmed A, Naus Claire, Nguyen Gabrielle, Castillo Jonathan, Ravindra Vijay M, Sutton Caitlin D, Weiner Howard L, Belfort Michael A (2025). Laparotomy-assisted, two-port fetoscopic myelomeningocele repair: infant to preschool outcomes.. Journal of neurosurgery. Pediatrics.

[81] Belfort Michael A, Whitehead William E, Shamshirsaz Alireza A, Bateni Zhoobin H, Olutoye Oluyinka O, Olutoye Olutoyin A, Mann David G, Espinoza Jimmy, Williams Erin, Lee Timothy C, Keswani Sundeep G, Ayres Nancy, Cassady Christopher I, Mehollin-Ray Amy R, Sanz Cortes Magdalena, Carreras Elena, Peiro Jose L, Ruano Rodrigo, Cass Darrell L (2017). Fetoscopic Open Neural Tube Defect Repair: Development and Refinement of a Two-Port, Carbon Dioxide Insufflation Technique.. Obstetrics and gynecology.

[82] (2025). Fetoscopic NEOX Cord 1K® Spina Bifida Repair. National Library of Medicine. ClinicalTrials.gov..

[83] (2026). Cellular Therapy for In Utero Repair of Myelomeningocele - The CuRe Trial (CuRe). National Library of Medicine. ClinicalTrials.gov..

[84] UC Davis Health Children’s Hospital. The CuRe Trial.. UC Davis Health Children’s Hospital..

[85] Grivell Rosalie M, Andersen Chad, Dodd Jodie M (2014). Prenatal versus postnatal repair procedures for spina bifida for improving infant and maternal outcomes.. The Cochrane database of systematic reviews.

[86] Flanders Tracy M, Heuer Gregory G, Madsen Peter J, Buch Vivek P, Mackell Catherine M, Alexander Erin E, Moldenhauer Julie S, Zarnow Deborah M, Flake Alan W, Adzick N Scott (2020). Detailed Analysis of Hydrocephalus and Hindbrain Herniation After Prenatal and Postnatal Myelomeningocele Closure: Report From a Single Institution.. Neurosurgery.

[87] Munoz Jessian L, Kelling Emma, Johnson Rebecca M, Buskmiller Cara, Whitehead William E, Joyeux Luc, Donepudi Roopali V, Nassr Ahmed A, Belfort Michael A, Castillo Jonathan, Castillo Heidi, Cortes Magdalena Sanz (2025). Impact of Prenatal Repair for Fetal Myelomeningocele on Gastrointestinal Function.. The Journal of pediatrics.

[88] Moldenhauer Julie S, Soni Shelly, Rintoul Natalie E, Spinner Susan S, Khalek Nahla, Martinez-Poyer Juan, Flake Alan W, Hedrick Holly L, Peranteau William H, Rendon Norma, Koh Jamie, Howell Lori J, Heuer Gregory G, Sutton Leslie N, Johnson Mark P, Adzick N Scott (2015). Fetal myelomeningocele repair: the post-MOMS experience at the Children's Hospital of Philadelphia.. Fetal diagnosis and therapy.

[89] Vonzun Ladina, Ruegg Ladina, Zepf Julia, Strübing Nele, Grehten Patrice, Meuli Martin, Mazzone Luca, Moehrlen Ueli, Ochsenbein-Koelble Nicole (2024). Middle Cerebral Artery Doppler before and after Fetal Spina Bifida Repair: An Indirect Sign of Hindbrain Compression and Decompression?. Fetal Diagnosis and Therapy.

[90] Danzer Enrico, Gerdes Marsha, Bebbington Michael W, Koh Jamie, Adzick Scott N, Johnson Mark P (2011). Fetal myelomeningocele surgery: preschool functional status using the Functional Independence Measure for children (WeeFIM).. Child's nervous system : ChNS : official journal of the International Society for Pediatric Neurosurgery.

[91] Danzer Enrico, Thomas Nina H, Thomas Allison, Friedman Karen B, Gerdes Marsha, Koh Jamie, Adzick N Scott, Johnson Mark P (2016). Long-term neurofunctional outcome, executive functioning, and behavioral adaptive skills following fetal myelomeningocele surgery.. American journal of obstetrics and gynecology.

[92] Danzer Enrico, Gerdes Marsha, Bebbington Michael W, Zarnow Deborah M, Adzick N Scott, Johnson Mark P (2010). Preschool neurodevelopmental outcome of children following fetal myelomeningocele closure.. American journal of obstetrics and gynecology.

[93] Johnson Mark Paul, Gerdes Marsha, Rintoul Natalie, Pasquariello Patrick, Melchionni Jeanne, Sutton Leslie N, Adzick N Scott (2006). Maternal-fetal surgery for myelomeningocele: neurodevelopmental outcomes at 2 years of age.. American journal of obstetrics and gynecology.

[94] Goodnight William H, Bahtiyar Ozan, Bennett Kelly A, Emery Stephen P, Lillegard J B, Fisher Allan, Goldstein Ruth, Jatres Jillian, Lim Foong-Yen, McCullough Laurence, Moehrlen Ueli, Moldenhauer Julie S, Moon-Grady Anita J, Ruano Rodrigo, Skupski Daniel W, Thom Elizabeth, Treadwell Marjorie C, Tsao KuoJen, Wagner Amy J, Waqar Lindsay N, Zaretsky Michael (2019). Subsequent pregnancy outcomes after open maternal-fetal surgery for myelomeningocele.. American journal of obstetrics and gynecology.

[95] Johnson Mark P, Bennett Kelly A, Rand Larry, Burrows Pamela K, Thom Elizabeth A, Howell Lori J, Farrell Jody A, Dabrowiak Mary E, Brock John W, Farmer Diana L, Adzick N Scott (2016). The Management of Myelomeningocele Study: obstetrical outcomes and risk factors for obstetrical complications following prenatal surgery.. American journal of obstetrics and gynecology.

[96] Wilson R Douglas, Johnson Mark P, Flake Alan W, Crombleholme Timothy M, Hedrick Holly L, Wilson Jordan, Adzick N Scott (2004). Reproductive outcomes after pregnancy complicated by maternal-fetal surgery.. American journal of obstetrics and gynecology.

[97] Wilson R Douglas, Lemerand Kerrie, Johnson Mark P, Flake Alan W, Bebbington Michael, Hedrick Holly L, Adzick N Scott (2010). Reproductive outcomes in subsequent pregnancies after a pregnancy complicated by open maternal-fetal surgery (1996-2007).. American journal of obstetrics and gynecology.

[98] Brock John W, Thomas John C, Baskin Laurence S, Zderic Stephen A, Thom Elizabeth A, Burrows Pamela K, Lee Hanmin, Houtrow Amy J, MacPherson Cora, Adzick N Scott (2019). Effect of Prenatal Repair of Myelomeningocele on Urological Outcomes at School Age.. The Journal of urology.

[99] Parizi João Luiz Gomes, Leal da Cruz Marcela, Andrade Maria Cristina, Garrone Gilmar, Ottoni Sérgio Leite, Cavalheiro Sérgio, Moron Antonio, Macedo Antonio (2020). A Comparative Analysis of Bladder Pattern of Patients who Underwent In Utero Versus Postnatal Myelomeningocele Repair.. The Journal of urology.

[100] Evangelista Aleksandra, Ruccolo Luigi, Friuli Valeria, Benazzo Marco, Conti Bice, Pisani Silvia (2026). Advances in Fetal Repair of Spina Bifida Integrating Prenatal Surgery, Stem Cells, and Biomaterials.. Biomedicines.

[101] Moldenhauer Julie S, Soni Shelly, Jatres Jillian, Gebb Juliana, Khalek Nahla, Paidas Teefey Christina, Johnson Mark P, Flake Alan W, Hedrick Holly L, Peranteau William H, Heuer Gregory G, Adzick N Scott (2020). Open Fetal Surgical Outcomes for Myelomeningocele Closure Stratified by Maternal Body Mass Index in a Large Single-Center Cohort.. Fetal diagnosis and therapy.

[102] Yamashiro Kaeli J, Farmer Diana L (2021). Fetal myelomeningocele repair: a narrative review of the history, current controversies and future directions.. Translational pediatrics.

[103] Jouannic Jean-Marie, Dugas Anaïs, Maurice Paul, Dhombres Ferdinand, Garel Catherine, Blondiaux Éléonore, Denis Timothée de Saint, Guilbaud Lucie (2026). Should we modify eligibility criteria for fetal surgery for open spinal dysraphism?. European journal of obstetrics, gynecology, and reproductive biology.

[104] Flanders Tracy M, Madsen Peter J, Pisapia Jared M, Hudgins Eric D, Mackell Catherine M, Alexander Erin E, Moldenhauer Julie S, Zarnow Deborah M, Flake Alan W, Adzick N Scott, Heuer Gregory G (2020). Improved Postoperative Metrics with Modified Myofascial Closure in Fetal Myelomeningocele Repair.. Operative neurosurgery (Hagerstown, Md.).

[105] Krause Matthias, Leibnitz Florian, Knüpfer Matthias Manfred, Merkenschlager Andreas, Griessenauer Christoph J, Gburek-Augustat Janina (2025). The potential impact of intraoperative neurophysiological monitoring on neurological function outcomes after postnatal spina bifida repair.. Child's nervous system : ChNS : official journal of the International Society for Pediatric Neurosurgery.

[106] Keil Corinna, Sass Benjamin, Schulze Maximilian, Köhler Siegmund, Axt-Fliedner Roland, Bedei Ivonne (2025). The Intrauterine Treatment of Open Spinal Dysraphism.. Deutsches Arzteblatt international.

[107] Zargarzadeh Nikan, Sambatur Enaja, Abiad May, Rojhani Ehsan, Javinani Ali, Northam Weston, Chmait Ramen H, Krispin Eyal, Aagaard Kjersti, Shamshirsaz Alireza A (2025). Gestational age at birth varies by surgical technique in prenatal open spina bifida repair: a systematic review and meta-analysis.. American journal of obstetrics and gynecology.

[108] Chmait Ramen H, Monson Martha A, Pham Huyen Q, Chu Jason K, Van Speybroeck Alexander, Chon Andrew H, Kontopoulos Eftichia V, Quintero Ruben A (2022). Percutaneous/mini-laparotomy fetoscopic repair of open spina bifida: a novel surgical technique.. American journal of obstetrics and gynecology.

[109] Danzer Enrico, Joyeux Luc, Flake Alan W, Deprest Jan (2020). Fetal surgical intervention for myelomeningocele: lessons learned, outcomes, and future implications.. Developmental medicine and child neurology.

[110] Stevenson Charles B, Fletcher Stephen, Larrew Thomas, Chu Jason K (2025). In-utero repair of open neural tube defects, lesion closure techniques and the choice of patch.. Best practice & research. Clinical obstetrics & gynaecology.

[111] Pedreira Denise A L, Zanon Nelci, de Sá Renato A M, Acacio Gregório L, Ogeda Edilson, Belem Teresa M L O U, Chmait Ramen H, Kontopoulos Eftichia, Quintero Ruben A (2014). Fetoscopic single-layer repair of open spina bifida using a cellulose patch: preliminary clinical experience.. The journal of maternal-fetal & neonatal medicine : the official journal of the European Association of Perinatal Medicine, the Federation of Asia and Oceania Perinatal Societies, the International Society of Perinatal Obstetricians.

[112] Naus Claire A, Mann David G, Andropoulos Dean B, Belfort Michael A, Sanz-Cortes Magdalena, Whitehead William E, Sutton Caitlin D (2025). "This is how we do it" Maternal and fetal anesthetic management for fetoscopic myelomeningocele repairs: the Texas Children's Fetal Center protocol.. International journal of obstetric anesthesia.

[113] Sanz Cortes Magdalena, Chmait Ramen H, Lapa Denise A, Belfort Michael A, Carreras Elena, Miller Jena L, Brawura Biskupski Samaha Robert, Sepulveda Gonzalez Gerardo, Gielchinsky Yuval, Yamamoto Masami, Persico Nicola, Santorum Marta, Otaño Lucas, Nicolaou Ermos, Yinon Yoav, Faig-Leite Fernanda, Brandt Reynaldo, Whitehead William, Maiz Nerea, Baschat Ahmet, Kosinski Przemyslaw, Nieto-Sanjuanero Adriana, Chu Jason, Kershenovich Amir, Nicolaides Kypros H (2021). Experience of 300 cases of prenatal fetoscopic open spina bifida repair: report of the International Fetoscopic Neural Tube Defect Repair Consortium.. American journal of obstetrics and gynecology.

[114] Sanz Cortes M, Lapa D A, Acacio G L, Belfort M, Carreras E, Maiz N, Peiro J L, Lim F Y, Miller J, Baschat A, Sepulveda G, Davila I, Gielchinsky Y, Benifla M, Stirnemann J, Ville Y, Yamamoto M, Figueroa H, Simpson L, Nicolaides K H (2019). Proceedings of the First Annual Meeting of the International Fetoscopic Myelomeningocele Repair Consortium.. Ultrasound in obstetrics & gynecology : the official journal of the International Society of Ultrasound in Obstetrics and Gynecology.

[115] Kunpalin Y., Karadjole V. S., Medeiros E. S. B., Domínguez‐Moreno M., Sichitiu J., Abbasi N., Ryan G., Shinar S., Snelgrove J. W., Kulkarni A. V., Van Mieghem T. (2025). Benefits and complications of fetal and postnatal surgery for open spina bifida: systematic review and proportional meta‐analysis. Ultrasound in Obstetrics &amp; Gynecology.

[116] Kabagambe Sandra K., Jensen Guy W., Chen Yue Julia, Vanover Melissa A., Farmer Diana L. (2017). Fetal Surgery for Myelomeningocele: A Systematic Review and Meta-Analysis of Outcomes in Fetoscopic versus Open Repair. Fetal Diagnosis and Therapy.

[117] Fareed Areeba, Farhat Solay, Kerhani Abed AlRazzak, Choudhary Anood, Raza Syeda Sadia Masood (2024). Fetal in-utero management of myelomeningocele: a mini-review on history, challenges, management gap, and recommendations. Annals of Medicine &amp; Surgery.

[118] Paslaru Francesca Gabriela, Panaitescu Anca Maria, Iancu George, Veduta Alina, Gica Nicolae, Paslaru Alexandru Catalin, Gheorghiu Anamaria, Peltecu Gheorghe, Gorgan Radu Mircea (2021). Myelomeningocele Surgery over the 10 Years Following the MOMS Trial: A Systematic Review of Outcomes in Prenatal versus Postnatal Surgical Repair. Medicina.

[119] Lapa (Pedreira) D. A., Acacio G. L., Gonçalves R. T., Sá R. A. M., Brandt R. A., Chmait R. H., Kontopoulos E. V., Quintero R. A. (2018). Percutaneous fetoscopic closure of large open spina bifida using a bilaminar skin substitute. Ultrasound in Obstetrics &amp; Gynecology.

[120] Chmait Ramen H., Chu Jason K., Van Speybroeck Alexander L., Llanes Ms. Arlyn S., Korst Lisa M., Nguyen HaiThuy N., Kontopoulos Eftichia V., Quintero Rubén A. (2025). Fetoscopic repair of open spina bifida between 26 0/7 and 27 6/7 gestational weeks and postnatal cerebrospinal fluid diversion. The Journal of Maternal-Fetal &amp; Neonatal Medicine.

[121] Corroenne Romain, Rangwani Sabrina, Whitehead William E., Johnson Rebecca M., Nassr Ahmed A., Buskmiller Cara, Munoz Jessian L., Castillo Jonathan, Castillo Heidi, Donepudi Roopali V., Belfort Michael A., Sanz Cortes Magdalena (2025). Neurodevelopmental Outcomes after Fetoscopic Myelomeningocele Repair. The Journal of Pediatrics.

[122] Kohn Jaden R., Rao Vibha, Sellner Allison A., Sharhan Dina, Espinoza Jimmy, Shamshirsaz Alireza A., Whitehead William E., Belfort Michael A., Sanz Cortes Magdalena (2018). Management of Labor and Delivery After Fetoscopic Repair of an Open Neural Tube Defect. Obstetrics &amp; Gynecology.

[123] Miranda Márcio Lopes, Ximenes Renato, Andrade Kleber Cursino, Baldo Carlos, Villarreal Mauro, Caetano Marcos Roberto, Lajos Giuliane, Dal Fabbro Mateus, Bustorff-Silva Joaquim Murray, Aydin Emrah, Peiro Jose L. (2025). Safety and Effectiveness of Fetal Myelomeningocele Repair: Case Series Analysis Using an Exteriorized Uterus and a Fetoscopic Approach. Fetal Diagnosis and Therapy.

[124] Sanz Cortes M., Davila I., Torres P., Yepez M., Lee W., Guimaraes C. V., Sangi‐Haghpeykar H., Whitehead W. E., Castillo J., Nassr A. A., Espinoza J., Shamshirsaz A. A., Belfort M. A. (2019). Does fetoscopic or open repair for spina bifida affect fetal and postnatal growth?. Ultrasound in Obstetrics &amp; Gynecology.

[125] Sanz Cortes M., Corroenne R., Pyarali M., Johnson R. M., Whitehead W. E., Espinoza J., Donepudi R., Castillo J., Castillo H., Mehollin‐Ray A. R., Shamshirsaz A. A., Nassr A. A., Belfort M. A. (2024). Ambulation after <i>in‐utero</i> fetoscopic or open neural tube defect repair: predictors for ambulation at 30 months. Ultrasound in Obstetrics &amp; Gynecology.

[126] Duron Vincent, Miller Russell, Feldstein Neil, Schmoke Nicholas, Wu Yeu Sanz, Shirel Tyler, Ring Laurence, Landau Ruth, Azizi Hana, Ingrassia Rosalie, Breslin Noelle, Simpson Lynn (2025). Outcomes Following Fetoscopic Repair of Myelomeningocele: A Prospective Single‐Center Experience. Prenatal Diagnosis.

[127] (2017). Committee Opinion No. 720: Maternal–Fetal Surgery for Myelomeningocele. Obstetrics &amp; Gynecology.

[128] Giné C., Arévalo S., Maíz N., Rodó C., Manrique S., Poca A., Molino J. A., Carreras E., López M. (2018). Fetoscopic two‐layer closure of open neural tube defects. Ultrasound in Obstetrics &amp; Gynecology.

[129] Bowman Robin, Alhajjat Amir, Muller Rya, Scoville Jonathan, Shaaban Aimen (2025). Achieving water-tight open spina bifida closure through a novel three-port three-layer fetoscopic repair. American Journal of Obstetrics &amp; Gynecology MFM.

[130] Cruz Stephanie M., Hameedi Sophia, Sbragia Lourenco, Ogunleye Oluseyi, Diefenbach Karen, Isaacs Albert M., Etchegaray Adolfo, Olutoye Oluyinka O. (2025). Fetoscopic Myelomeningocele (MMC) Repair: Evolution of the Technique and a Call for Standardization. Journal of Clinical Medicine.

[131] (2023). MOMS3: Follow-up in the Teen and Young Adult Years to the Management of Myelomeningocele Study. Children's Hospital of Philadelphia.

[132] Dhaulakhandi Dhara B., Rohilla Seema, Rattan Kamal Nain (2010). Neural Tube Defects: Review of Experimental Evidence on Stem Cell Therapy and Newer Treatment Options. Fetal Diagnosis and Therapy.

[133] Watanabe Miho, Kim Aimee G., Flake Alan W. (2014). Tissue Engineering Strategies for Fetal Myelomeningocele Repair in Animal Models. Fetal Diagnosis and Therapy.

[134] Winkler Sally M., Harrison Michael R., Messersmith Phillip B. (2019). Biomaterials in fetal surgery. Biomaterials Science.

[135] Fauza Dario O., Jennings Russell W., Teng Yang D., Snyder Evan Y. (2008). Neural stem cell delivery to the spinal cord in an ovine model of fetal surgery for spina bifida. Surgery.

[136] Lankford Lee, Chen Y. Julia, Saenz Zoe, Kumar Priyadarsini, Long Connor, Farmer Diana, Wang Aijun (2017). Manufacture and preparation of human placenta-derived mesenchymal stromal cells for local tissue delivery. Cytotherapy.

[137] Jackson Jordan E., Pivetti Christopher, Stokes Sarah C., Theodorou Christina M., Kumar Priyadarsini, Paxton Zachary J., Hyllen Alicia, Reynaga Lizette, Wang Aijun, Farmer Diana L. (2021). Placental Mesenchymal Stromal Cells: Preclinical Safety Evaluation for Fetal Myelomeningocele Repair. Journal of Surgical Research.

[138] Theodorou Christina M., Stokes Sarah C., Jackson Jordan E., Pivetti Christopher D., Kumar Priyadarsini, Yamashiro Kaeli J., Paxton Zachary J., Reynaga Lizette, Hyllen Alicia A., Wang Aijun, Farmer Diana L. (2022). Efficacy of clinical-grade human placental mesenchymal stromal cells in fetal ovine myelomeningocele repair. Journal of Pediatric Surgery.

[139] Stokes Sarah C, Theodorou Christina M, Jackson Jordan E, Pivetti Christopher, Kumar Priyadarsini, Yamashiro Kaeli J, Paxton Zachary J, Reynaga Lizette, Hyllen Alicia, Wang Aijun, Farmer Diana L (2022). Long-term safety evaluation of placental mesenchymal stromal cells for in utero repair of myelomeningocele in a novel ovine model. Journal of Pediatric Surgery.

[140] Wang Aijun, Brown Erin G., Lankford Lee, Keller Benjamin A., Pivetti Christopher D., Sitkin Nicole A., Beattie Michael S., Bresnahan Jacqueline C., Farmer Diana L. (2015). Placental Mesenchymal Stromal Cells Rescue Ambulation in Ovine Myelomeningocele. Stem Cells Translational Medicine.

[141] Vanover Melissa, Pivetti Christopher, Lankford Lee, Kumar Priyadarsini, Galganski Laura, Kabagambe Sandra, Keller Benjamin, Becker James, Chen Y. Julia, Chung Karen, Lee Chelsey, Paxton Zachary, Deal Bailey, Goodman Laura, Anderson Jamie, Jensen Guy, Wang Aijun, Farmer Diana (2019). High density placental mesenchymal stromal cells provide neuronal preservation and improve motor function following in utero treatment of ovine myelomeningocele. Journal of Pediatric Surgery.

[142] Kabagambe Sandra, Keller Benjamin, Becker James, Goodman Laura, Pivetti Christopher, Lankford Lee, Chung Karen, Lee Chelsey, Chen Y. Julia, Kumar Priyadarsini, Vanover Melissa, Wang Aijun, Farmer Diana (2018). Placental mesenchymal stromal cells seeded on clinical grade extracellular matrix improve ambulation in ovine myelomeningocele. Journal of Pediatric Surgery.

[143] Galganski Laura A, Kumar Priyadarsini, Vanover Melissa A, Pivetti Christopher D, Anderson Jamie E, Lankford Lee, Paxton Zachary J, Chung Karen, Lee Chelsey, Hegazi Mennatalla S, Yamashiro Kaeli J, Wang Aijun, Farmer Diana L (2020). In utero treatment of myelomeningocele with placental mesenchymal stromal cells — Selection of an optimal cell line in preparation for clinical trials. Journal of Pediatric Surgery.

[144] CuRe Trial Animation. ProLifedoc Inc.

[145] Lazow Stefanie P., Fauza Dario O. (2019). Transamniotic Stem Cell Therapy. Advances in Experimental Medicine and Biology.

[146] Shieh Hester F, Tracy Sarah A, Hong Charles R, Chalphin Alexander V, Ahmed Azra, Rohrer Lucas, Zurakowski David, Fauza Dario O (2019). Transamniotic stem cell therapy (TRASCET) in a rabbit model of spina bifida. Journal of Pediatric Surgery.

[147] Dionigi Beatrice, Ahmed Azra, Brazzo Joseph, Connors John Patrick, Zurakowski David, Fauza Dario O. (2015). Partial or complete coverage of experimental spina bifida by simple intra-amniotic injection of concentrated amniotic mesenchymal stem cells. Journal of Pediatric Surgery.

[148] Dionigi Beatrice, Brazzo Joseph A., Ahmed Azra, Feng Christina, Wu Yaotang, Zurakowski David, Fauza Dario O. (2015). Trans-amniotic stem cell therapy (TRASCET) minimizes Chiari-II malformation in experimental spina bifida. Journal of Pediatric Surgery.

[149] Feng Christina, D. Graham Christopher, Connors John Patrick, Brazzo Joseph, Zurakowski David, Fauza Dario O. (2016). A comparison between placental and amniotic mesenchymal stem cells for transamniotic stem cell therapy (TRASCET) in experimental spina bifida. Journal of Pediatric Surgery.

[150] Li Xiaoshuai, Yuan Zhengwei, Wei Xiaowei, Li Hui, Zhao Guifeng, Miao Jiaoning, Wu Di, Liu Bo, Cao Songying, An Dong, Ma Wei, Zhang Henan, Wang Weilin, Wang Qiushi, Gu Hui (2016). Application potential of bone marrow mesenchymal stem cell (BMSCs) based tissue-engineering for spinal cord defect repair in rat fetuses with spina bifida aperta. Journal of Materials Science: Materials in Medicine.

[151] Ma Wei, Wei Xiaowei, Gu Hui, Li Hui, Guan Kaoping, Liu Dan, Chen Lizhu, Cao Songying, An Dong, Zhang Henan, Huang Tianchu, Miao Jianing, Zhao Guifeng, Wu Di, Liu Bo, Wang Weilin, Yuan Zhengwei (2015). Sensory neuron differentiation potential of in utero mesenchymal stem cell transplantation in rat fetuses with spina bifida aperta. Birth Defects Research Part A: Clinical and Molecular Teratology.

[152] Saadai Payam, Wang Aijun, Nout Yvette S., Downing Timothy L., Lofberg Katrine, Beattie Michael S., Bresnahan Jacqueline C., Li Song, Farmer Diana L. (2013). Human induced pluripotent stem cell-derived neural crest stem cells integrate into the injured spinal cord in the fetal lamb model of myelomeningocele. Journal of Pediatric Surgery.

[153] Basler Michelle, Pontiggia Luca, Biedermann Thomas, Reichmann Ernst, Meuli Martin, Mazzone Luca (2019). Bioengineering of Fetal Skin: Differentiation of Amniotic Fluid Stem Cells into Keratinocytes. Fetal Diagnosis and Therapy.

[154] Mazzone Luca, Moehrlen Ueli, Ochsenbein‐Kölble Nicole, Pontiggia Luca, Biedermann Thomas, Reichmann Ernst, Meuli Martin (2019). Bioengineering and in utero transplantation of fetal skin in the sheep model: A crucial step towards clinical application in human fetal spina bifida repair. Journal of Tissue Engineering and Regenerative Medicine.

[155] Watanabe Miho, Jo Jun-ichiro, Radu Antonetta, Kaneko Michio, Tabata Yasuhiko, Flake Alan W. (2010). A Tissue Engineering Approach for Prenatal Closure of Myelomeningocele with Gelatin Sponges Incorporating Basic Fibroblast Growth Factor. Tissue Engineering Part A.

[156] Watanabe Miho, Li Hiaying, Roybal Jessica, Santore Matthew, Radu Antonetta, Jo Jun-Ichiro, Kaneko Michio, Tabata Yasuhiko, Flake Alan (2011). A Tissue Engineering Approach for Prenatal Closure of Myelomeningocele: Comparison of Gelatin Sponge and Microsphere Scaffolds and Bioactive Protein Coatings. Tissue Engineering Part A.

[157] Watanabe Miho, Li Haiying, Kim Aimee G., Weilerstein Aaron, Radu Anteneta, Davey Marcus, Loukogeorgakis Stavros, Sánchez Melissa D., Sumita Kazutaka, Morimoto Naoki, Yamamoto Masaya, Tabata Yasuhiko, Flake Alan W. (2016). Complete tissue coverage achieved by scaffold-based tissue engineering in the fetal sheep model of Myelomeningocele. Biomaterials.

[158] Farrelly James S, Bianchi Anthony H, Ricciardi Adele S, Buzzelli Gina L, Ahle Samantha L, Freedman-Weiss Mollie R, Luks Valerie L, Saltzman W Mark, Stitelman David H (2019). Alginate microparticles loaded with basic fibroblast growth factor induce tissue coverage in a rat model of myelomeningocele. Journal of Pediatric Surgery.

[159] Eggink A.J., Roelofs L.A.J., Feitz W.F.J., Wijnen R.M.H., Mullaart R.A., Grotenhuis J.A., van Kuppevelt T.H., Lammens M.M.Y., Crevels A.J., Hanssen A., van den Berg P.P. (2005). In utero Repair of an Experimental Neural Tube Defect in a Chronic Sheep Model Using Biomatrices. Fetal Diagnosis and Therapy.

[160] Eggink A.J., Roelofs L.A.J., Lammens M.M.Y., Feitz W.F.J., Wijnen R.M.H., Mullaart R.A., van Moerkerk H.T.B., van Kuppevelt T.H., Crevels A.J., Hanssen A., Lotgering F.K., van den Berg P.P. (2006). Histological Evaluation of Acute Covering of an Experimental Neural Tube Defect with Biomatrices in Fetal Sheep. Fetal Diagnosis and Therapy.

[161] Eggink A.J., Roelofs L.A.J., Feitz W.F.J., Wijnen R.M.H., Lammens M.M.Y., Mullaart R.A., van Moerkerk H.T.B., van Kuppevelt T.H., Crevels A.J., Verrijp K., Lotgering F.K., van den Berg P.P. (2007). Delayed Intrauterine Repair of an Experimental Spina Bifida with a Collagen Biomatrix. Pediatric Neurosurgery.

[162] Fontecha Cesar G., Peiro Jose L., Sevilla Juan J., Aguirre Marius, Soldado Francisco, Fresno Laura, Fonseca Carla, Chacaltana Asiul, Martinez Vicente (2011). Fetoscopic coverage of experimental myelomeningocele in sheep using a patch with surgical sealant. European Journal of Obstetrics &amp; Gynecology and Reproductive Biology.

[163] Fontecha Cesar G., Peiro Jose L., Aguirre Marius, Soldado Francisco, Añor Sonia, Fresno Laura, Martinez-Ibañez Vicente (2009). Inert patch with bioadhesive for gentle foetal surgery of myelomeningocele in a sheep model. European Journal of Obstetrics &amp; Gynecology and Reproductive Biology.

[164] Peiro Jose L., Fontecha Cesar G., Ruano Rodrigo, Esteves Marielle, Fonseca Carla, Marotta Mario, Haeri Sina, Belfort Michael A. (2013). Single-Access Fetal Endoscopy (SAFE) for myelomeningocele in sheep model I: amniotic carbon dioxide gas approach. Surgical Endoscopy.

[165] Brown Erin G., Saadai Payam, Pivetti Christopher D., Beattie Michael S., Bresnahan Jacqueline C., Wang Aijun, Farmer Diana L. (2014). In utero repair of myelomeningocele with autologous amniotic membrane in the fetal lamb model. Journal of Pediatric Surgery.

[166] Saadai Payam, Nout Yvette S., Encinas Jose, Wang Aijun, Downing Timothy L., Beattie Michael S., Bresnahan Jacqueline C., Li Song, Farmer Diana L. (2011). Prenatal repair of myelomeningocele with aligned nanofibrous scaffolds—a pilot study in sheep. Journal of Pediatric Surgery.

[167] Oliveira Rita de Cássia Sanchez e, Valente Paulo Roberto, Abou-Jamra Rogério C., Araújo Andrezza, Saldiva Paulo Hilário, Pedreira Denise Araújo Lapa (2007). Biosynthetic cellulose induces the formation of a neoduramater following pre-natal correction of meningomyelocele in fetal sheep. Acta Cirurgica Brasileira.

[168] Herrera Silvia Rejane Fontoura, Leme Ricardo José de Almeida, Valente Paulo Roberto, Caldini Elia Garcia, Saldiva Paulo Hilário Nascimento, Pedreira Denise Araujo Lapa (2012). Comparison between two surgical techniques for prenatal correction of meningomyelocele in sheep.. Einstein (Sao Paulo, Brazil).

[169] Kunpalin Yada, Vergote Simen, Joyeux Luc, Telli Onur, David Anna L., Belfort Michael, De Coppi Paolo, Deprest Jan (2023). Local host response of commercially available dural patches for fetal repair of spina bifida aperta in rabbit model. Prenatal Diagnosis.

[170] Dasargyri Athanasia, Reichmann Ernst, Moehrlen Ueli (2019). Bio-engineering of fetal cartilage for in utero spina bifida repair. Pediatric Surgery International.

[171] Gansevoort Merel, Oostendorp Corien, Bouwman Linde F., Tiemessen Dorien M., Geutjes Paul J., Feitz Wout F. J., van Kuppevelt Toin H., Daamen Willeke F. (2024). Collagen-Heparin-FGF2-VEGF Scaffolds Induce a Regenerative Gene Expression Profile in a Fetal Sheep Wound Model. Tissue Engineering and Regenerative Medicine.

[172] Snowise Saul, Mann Lovepreet, Morales Yisel, Moise Kenneth J., Johnson Anthony, Fletcher Stephen, Grill Raymond J., Tseng Scheffer C.G., Papanna Ramesha (2017). Cryopreserved human umbilical cord versus biocellulose film for prenatal spina bifida repair in a physiologic rat model. Prenatal Diagnosis.

[173] Mann Lovepreet K., Won Jong Hak, Patel Rajan, Bergh Eric P., Garnett Jeannine, Bhattacharjee Meenakshi B., Narayana Ponnada A., Jain Ranu, Fletcher Stephen A., Lai Dejian, Papanna Ramesha (2021). Allografts for Skin Closure during In Utero Spina Bifida Repair in a Sheep Model. Journal of Clinical Medicine.

[174] Papanna R., Moise K. J., Mann L. K., Fletcher S., Schniederjan R., Bhattacharjee M. B., Stewart R. J., Kaur S., Prabhu S. P., Tseng S. C. G. (2016). Cryopreserved human umbilical cord patch for in‐utero spina bifida repair. Ultrasound in Obstetrics &amp; Gynecology.

[175] Papanna Ramesha, Mann Lovepreet, Snowise Saul, Morales Yisel, Prabhu Sanjay, Tseng Scheffer, Grill Raymond, Fletcher Stephen, Moise Kenneth (2016). Neurological Outcomes after Human Umbilical Cord Patch for In Utero Spina Bifida Repair in a Sheep Model. American Journal of Perinatology Reports.

[176] Athiel Y, Jouannic J-M, Mauffré, Dehan C, Adam C, Blot S, Lallemant P, De Saint Denis T, Larghero J, Nasone J, Guilbaud L (2025). Allogenic Umbilical Cord-Derived Mesenchymal Stromal Cells Improve Motor Function in Prenatal Surgical Repair of Myelomeningocele: An Ovine Model Study. Obstetric Anesthesia Digest.

[177] Guilbaud Lucie, Dugas Anaïs, Weber Mathilde, Deflers Carole, Lallemant Pauline, Lilin Thomas, Adam Clovis, Cras Audrey, Mebarki Miryam, Zérah Michel, Faivre Lionel, Larghero Jérôme, Jouannic Jean-Marie (2022). In utero treatment of myelomeningocele with allogenic umbilical cord-derived mesenchymal stromal cells in an ovine model. Current Research in Translational Medicine.

[178] Papanna Ramesha, Fletcher Stephen, Moise Kenneth J., Mann Lovepreet K., Tseng Scheffer C. G. (2016). Cryopreserved Human Umbilical Cord for In Utero Myeloschisis Repair. Obstetrics &amp; Gynecology.

[179] Meuli Martin, Meuli-Simmen Claudia, Mazzone Luca, Tharakan Sasha J., Zimmermann Roland, Ochsenbein Nicole, Moehrlen Ueli (2017). In utero Plastic Surgery in Zurich: Successful Use of Distally Pedicled Random Pattern Transposition Flaps for Definitive Skin Closure during Open Fetal Spina Bifida Repair. Fetal Diagnosis and Therapy.

[180] Berris M, Shoham M (2006). Febotics – a marriage of fetal surgery and robotics. Computer Aided Surgery.

[181] Aaronson Oran S., Tulipan Noel B., Cywes Robert, Sundell Håkan W., Davis Georges H., Bruner Joseph P., Richards William O. (2002). Robot-Assisted Endoscopic Intrauterine Myelomeningocele Repair: A Feasibility Study. Pediatric Neurosurgery.

[182] Knight Colin G., Lorincz Attila, Johnson Anthony, Gidell Kelly, Rabah Rajah, Klein Michael D., Langenburg Scott E. (2004). Robot-enhanced fetoscopic surgery. Journal of Pediatric Surgery.

[183] Kohl T., Hartlage M. G., Kiehitz D., Westphal M., Buller T., Achenbach S., Aryee S., Gembruch U., Brentrup A. (2003). Percutaneous fetoscopic patch coverage of experimental lumbosacral full-thickness skin lesions in sheep. Surgical Endoscopy.

[184] Gervasoni Simone, Lussi Jonas, Viviani Silvia, Boehler Quentin, Ochsenbein Nicole, Moehrlen Ueli, Nelson Bradley J. (2022). Magnetically Assisted Robotic Fetal Surgery for the Treatment of Spina Bifida. IEEE Transactions on Medical Robotics and Bionics.

[185] Kunpalin Yada, Kik Charlotte C., Lebouthillier Francis, Abbasi Nimrah, Ryan Greg, Spoor Jochem, Looi Thomas, Kulkarni Abhaya V., Van Mieghem Tim (2025). Fetoscopic Robotic Open Spina Bifida Treatment (<scp>FROST</scp>): A Preclinical Feasibility and Learning Curve Study. BJOG: An International Journal of Obstetrics &amp; Gynaecology.

[186] Biglino Giovanni, Milano Elena G, Capelli Claudio, Wray Jo, Shearn Andrew IU, Caputo Massimo, Bucciarelli-Ducci Chiara, Taylor Andrew M, Schievano Silvia (2019). Three-dimensional printing in congenital heart disease: Considerations on training and clinical implementation from a teaching session. The International Journal of Artificial Organs.

[187] Fils Aaron J., Kasmirski Julia, Okpaise Oluwateniayo, Reynolds John M., Tonni Gabriele, Werner Heron, Ruano Rodrigo (2024). The Use of 3D Printing in Fetal Surgery for Surgical Planning: A Scoping Review. Journal of Clinical Medicine.

[188] Miller J. L., Ahn E. S., Garcia J. R., Miller G. T., Satin A. J., Baschat A. A. (2018). Ultrasound‐based three‐dimensional printed medical model for multispecialty team surgical rehearsal prior to fetoscopic myelomeningocele repair. Ultrasound in Obstetrics &amp; Gynecology.

[189] Kik Charlotte C., Kunpalin Yada, Kulkarni Abhaya V., DeKoninck Philip L. J., Spoor Jochem K. H., Van Mieghem Tim (2025). Global variability in fetal spina bifida surgery: a survey of neurosurgical strategies. Journal of Neurosurgery: Pediatrics.

[190] Keil Corinna, Wiora Noemi, Krispin Eyal, Windhorst Anita, Axt‐Fliedner Roland, Bedei Ivonne (2025). Evolving Practices in Prenatal Open Spinal Dysraphism: A Global Survey of Selection Criteria, Surgical Techniques, and Diagnostic Trends. Prenatal Diagnosis.

[191] Nulens K., Kunpalin Y., Nijs K., Carvalho J. C. A., Pollard L., Abbasi N., Ryan G., Mieghem T. Van (2024). Enhanced recovery after fetal spina bifida surgery: global practice. Ultrasound in Obstetrics &amp; Gynecology.

[192] Gallagher Katie, Crombag Neeltje, Prashar Kavita, Deprest Jan, Ourselin Sebastien, David Anna L., Marlow Neil (2023). Global Policy and Practice for Intrauterine Fetal Resuscitation During Fetal Surgery for Open Spina Bifida Repair. JAMA Network Open.

[193] Castillo Jonathan, Locastro Mary M, Corroenne Romain, Malhotra Anjali, Van Speybroeck Alexander, Lai Grace, Belfort Michael A, Sanz Cortes Magdalena, Castillo Heidi (2025). Maternal–fetal surgery for myelomeningocele longitudinal follow-up model: Mitigation of care fragmentation through care coordination and outcomes reporting. Journal of Pediatric Rehabilitation Medicine.

[194] Kohl Thomas, Tchatcheva Kristina, Merz Waltraut, Wartenberg Hans C., Heep Axel, Müller Andreas, Franz Axel, Stressig Rüdiger, Willinek Winfried, Gembruch Ulrich (2008). Percutaneous fetoscopic patch closure of human spina bifida aperta: advances in fetal surgical techniques may obviate the need for early postnatal neurosurgical intervention. Surgical Endoscopy.

[195] Degenhardt J., Schürg R., Winarno A., Oehmke F., Khaleeva A., Kawecki A., Enzensberger C., Tinneberg H.‐R., Faas D., Ehrhardt H., Axt‐Fliedner R., Kohl T. (2014). Percutaneous minimal‐access fetoscopic surgery for spina bifida aperta. Part <scp>II</scp>: maternal management and outcome. Ultrasound in Obstetrics &amp; Gynecology.

[196] Kohl T. (2014). Percutaneous minimally invasive fetoscopic surgery for spina bifida aperta. Part I: surgical technique and perioperative outcome. Ultrasound in Obstetrics &amp; Gynecology.

[197] El Damaty Ahmed, Elsässer Michael, Pfeifer Ulrich, Kotzaeridou Urania, Gille Christian, Spratte Julia, Zivanovic Oliver, Sohn Christoph, Krieg Sandro M., Bächli Heidrun, Unterberg Andreas (2025). The first experience with 16 open microsurgical fetal surgeries for myelomeningocele in Germany. European Journal of Paediatric Neurology.

[198] Vonzun L, Kahr MK, Noll F, Mazzone L, Moehrlen U, Meuli M, Hüsler M, Krähenmann F, Zimmermann R, Ochsenbein‐Kölble N (2020). Systematic classification of maternal and fetal intervention‐related complications following open fetal myelomeningocele repair – results from a large prospective cohort. BJOG: An International Journal of Obstetrics &amp; Gynaecology.

[199] Feng Ming, Chen Pei-Chen, Lin Guan-Ru, Lin Tzu-Yi, Hsieh T'sang-T'ang, Shaw Steven W. (2024). The clinical experience of fetoscopic repair of myelomeningocele in Taiwan: The dilemma in prenatal decision-making and first successful case. Taiwanese Journal of Obstetrics and Gynecology.

[200] Pruthi Vagisha, Abbasi Nimrah, Ryan Greg, Drake James, Kulkarni Abhaya V., Kwan-Wong Terence, Phillips John, Thakur Varsha, Church Paige, Diambomba Yenge, Kelly Edmond, Vermeersch Leslie, Pollard Lindsay, Carvalho Jose C.A., Van Mieghem Tim (2021). Fetal Surgery for Open Spina Bifida in Canada: Initial Results. Journal of Obstetrics and Gynaecology Canada.

[201] Kik Charlotte C., Kunpalin Yada, Kulkarni Abhaya V., Varghese Abby, Abbasi Nimrah, Ryan Greg, Dekoninck Philip L. J., Church Paige T., Malhotra Armaan, Raghuram Kamini, Kelly Edmond, Van Mieghem Tim (2024). Contemporary Outcomes of a National Fetal Spina Bifida Surgery Service. Prenatal Diagnosis.

[202] Friszer S, Dhombres F, Di Rocco F, Rigouzzo A, Garel C, Guilbaud L (2016). Résultats préliminaires de l’étude PRIUM : programme de réparation in utero des myéloméningocèles. J Gynecol Obstet Biol Reprod(Paris).

[203] Guilbaud Lucie, Maurice Paul, Lallemant Pauline, De Saint-Denis Timothée, Maisonneuve Emeline, Dhombres Ferdinand, Friszer Stéphanie, Di Rocco Federico, Garel Catherine, Moutard Marie-Laure, Lachtar Mohamed-Ali, Rigouzzo Agnès, Forin Véronique, Zérah Michel, Jouannic Jean-Marie (2021). Open fetal surgery for myelomeningocele repair in France. Journal of Gynecology Obstetrics and Human Reproduction.

[204] Hadassah International. Hadassah Medical Center
Performs Israel’s First Spina Bifida Fetal Surgery to
Lessen Crippling Spinal Damage. Hadassah International.

[205] Garg Stuti P., Shah Krish V., Lentskevich Marina, Yau Alice, Gosain Arun K. (2024). Prenatal Spina Bifida Repair: A Survey of Current Practice in the United States. Plastic and Reconstructive Surgery - Global Open.

[206] Cavolo Alice, Gastmans Chris, Crombag Neeltje (2024). Ethical challenges in conducting maternal-fetal surgery trials. A systematic review. Pediatric Research.

[207] Austin Mary T., Cole Thomas R., McCullough Laurence B., Chervenak Frank A. (2019). Ethical challenges in invasive maternal-fetal intervention. Seminars in Pediatric Surgery.

[208] HARRISON MICHAEL R., ADZICK N. SCOTT (1991). The Fetus as a Patient Surgical Considerations. Annals of Surgery.

[209] Copel Joshua A. (1991). The Unborn Patient: Prenatal Diagnosis and Treatment. JAMA: The Journal of the American Medical Association.

[210] Harrison MR, Evans ML, Adzick NS, Holzgreve W (2001). The Unborn Patient: The Art and Science of Fetal Therapy.

